# Organic residue analysis reveals the function of bronze age metal daggers

**DOI:** 10.1038/s41598-022-09983-3

**Published:** 2022-04-12

**Authors:** Isabella Caricola, Alasdair Charles, Jacopo Tirillò, Fraser Charlton, Huw Barton, Francesco Breglia, Alberto Rossi, Maria Chiara Deflorian, Anna Maria De Marinis, Susanna Harris, Alessio Pellegrini, Federico Scacchetti, Paolo Boccuccia, Monica Miari, Andrea Dolfini

**Affiliations:** 1 School of History, Classics and Archaeology, Newcastle University, Armstrong Building, Newcastle Upon Tyne, NE1 7RU UK; 2 School of Engineering, Newcastle University, Newcastle Upon Tyne, NE1 7RU UK; 3 Dipartimento Ingegneria Chimica Materiali Ambiente, Sapienza University, 00185 Rome, Italy; 4 Cellular Pathology, Royal Victoria Infirmary, Newcastle Upon Tyne, NE1 4LP UK; 5 School of Archaeology and Ancient History, University of Leicester, Leicester, LE1 7RH UK; 6 Dipartimento di Beni Culturali, Salento University, 73100 Lecce, Italy; 7 Officina Temporis, 60026 Marche, Italy; 8 MUSE-Museo delle Scienze, 38122 Trento, Italy; 9 Istituto Superiore per la Protezione e la Ricerca Ambientale ISPRA, Ozzano dell’Emilia, 40064 Bologna, Italy; 10 School of Humanities, University of Glasgow, Glasgow, G12 8QQ Scotland; 11 Department of Classics, Sapienza University, 00185 Rome, Italy; 12 Archeosistemi, 42124 Reggio Emilia, Italy; 13 Ministero della Cultura, Museo delle Civiltà, 00144 Rome, Italy; 14 Soprintendenza Archeologia Belle Arti e Paesaggio per la Città Metropolitana di Bologna e le Province di Modena, Reggio Emilia e Ferrara, 40123 Bologna, Italy

**Keywords:** Biological techniques, Metals

## Abstract

The article discusses results of organic residue analysis performed on ten copper-alloy daggers from Bronze Age Pragatto, Italy, *c*.1550–1250 BCE. Metal daggers are widespread in Chalcolithic and Bronze Age Europe, yet their social and practical roles are still hotly debated. Are they symbolic or functional? Are they tools or weapons? How were they used? For what tasks and on what materials? The research addresses these questions through a novel application of biochemical staining and SEM–EDX analysis. The method has proved successful in extracting and identifying animal residues located on cutting edges including bone, muscle, and tendons. These are interpreted as evidence of prehistoric carcass butchering and carving. Further residues were observed on blade faces and hafting plates or tangs; these are interpreted as remnants of bone handles and sheaths, the latter made of either wood fibers or processed hide and fur. The readings proposed in the article are validated by original experiments with replica daggers, as detailed in the Supplementary Materials. The analysis and experiments shed new light on Bronze Age metal daggers, showing that they were fully functional tools (and perhaps tool-weapons) primarily utilized for the processing of animal carcasses. This original research result contributes significant knowledge towards interpreting an under-studied, yet socially salient, prehistoric metal artifact.

## Introduction

Daggers are ubiquitous yet poorly understood artifacts from prehistoric Europe. They first appeared near-simultaneously in eastern/central Europe, the Alps, and the Italian peninsula in the early 4th millennium BCE^1–4^. From the outset, daggers were made from either flint or copper (first alloyed with arsenic, and later with tin) depending on source proximity and cultural preferences. By the early 2nd millennium BCE, daggers were being made, used, and exchanged from Crete in the south to Scandinavia in the north, and from the Russian steppes in the east to Ireland in the west. After this cross-material *floruit*, flint and metal daggers parted ways, with the former all but disappearing from the archaeological record and the latter continuing to be made and used throughout the Bronze Age^5, 6^.

Early metal daggers were long thought to be non-functional insignia of male identity and power due to perceived weaknesses in design and alloy composition^7, 8^. Pioneering applications of metalwork wear analysis suggest that this might not be the case. Wall’s^9^ examination of 55 Early Bronze Age daggers from southern Britain, for example, indicates that protracted use, repairs, and curation were not uncommon. Similarly, Dolfini^10^ and Iaia and Dolfini^11^ noticed high rates of edge sharpening coupled with minor edge damage on 15 Chalcolithic daggers from Italy. They proposed that the damage might be due to contact with soft materials such as animal tissue. Generally, size reduction due to repeated sharpening is common on prehistoric metal daggers, denoting a preoccupation for keeping these objects sharp throughout their use lives^12, 13^.

None of these studies is conclusive due to their narrow regional and chronological samples. Even if deployed on larger assemblages, however, usewear analysis is unlikely to address broad questions concerning the function of early metal daggers due to (a) high rates of edge corrosion; (b) certain uses not leaving discernible traces; and (c) some of the traces being unspecific. Under these circumstances, insights into dagger uses can be provided by the organic residues trapped in the objects’ corroded surfaces, or by their imprints, which may reveal the substances they had been in contact with. Such an approach has long been applied to ancient stone, shell, and ceramic artifacts but never (to the best of our knowledge) to copper-alloy tools or weapons^14–22^.

In this paper, we discuss non-destructive organic residue analysis as performed on ten freshly excavated copper-alloy daggers from Pragatto, a Bronze Age domestic site in northern Italy. We present (a) the site and context; (b) results of qualitative residue analysis carried out on the daggers; (c) results of SEM–EDX analysis; and (d) details of the analytical method. We demonstrate that the research sheds new light on the uses of Bronze Age daggers. Importantly, the approach presented here can be replicated on other copper-alloy tools and weapons from world prehistory. It is therefore a significant addition to the analytical toolkit available to students of the human past.

## Archaeological context

Pragatto (Bologna, Italy) is a prehistoric settlement site excavated in 2016–2017^23^. The site is part of the broader *Terramare* settlement system, which characterized human occupation of the Po River valley, northern Italy, in the Middle and Late Bronze Age, *c*.1650–1200 BCE^24^. The *Terramare* system emerged in the early stages of the Middle Bronze Age due to combined demographic growth, likely population transfer from Alpine lake-side villages, and a novel ability to manage wet and riverine landscapes, which enabled large-scale crop cultivation of heavy alluvial soils^25, 26^.

*Terramare* sites are square villages ranging from 1 to 20 hectares in size. They were normally built near rivers or streams, whose courses were diverted to fill in the ditches surrounding the sites; embankments and palisades also encircled most sites^27, 28^. The *Terramare* settlement system provided a stable framework for sociopolitical organization in north-eastern Italy until the advanced Bronze Age. The system dramatically collapsed *c*.1200 BCE due to either climatic deterioration or political instability, and perhaps both^28, 29^.

**Figure 1:**
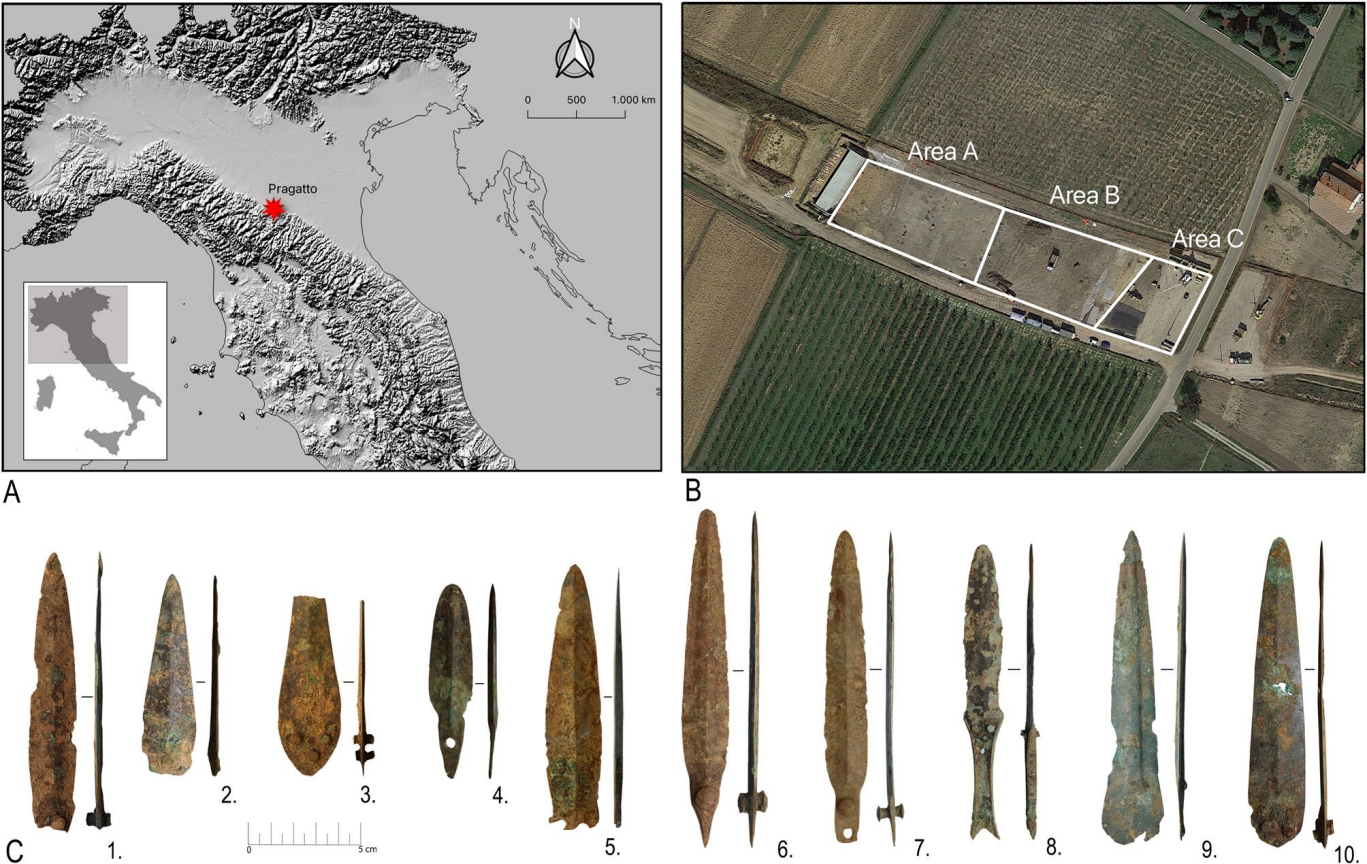
(**A**) Site location (the map was generated by I.C. through QGIS v.3.16, https://qgis.org); (**B**) Aerial view of the site highlighting excavation areas A, B and C (source: Google Earth); (**C**) Copper-alloy daggers analyzed as part of the research. Specimen (**1**) no 1617; (**2**) no 2037; (**3**) no 175; (**4**) no 1707; (**5**) no 2041; (**6**) no 1798; (**7**) no 2035; (**8**) no 1683; (**9**) no 1321; (**10**) no 264.

At Pragatto, controlled excavations covered a 6900 sqm area (Fig. 1A) corresponding to the southern portion of the Bronze Age village (Fig. 1B, areas A and B) and surrounding ditch and banks (Fig. 1B, area C). Site stratigraphy and cultural materials were found to be best preserved in area B thanks to a large fire that swept through the village in prehistoric times. Here, investigations revealed nine burnt-down houses, refuse pits, animal pens, and other features. Due to its excellent preservation, area B returned large numbers of portable finds including over 150 bronzes, e.g., daggers, arrowheads, and craft tools of various descriptions^23, 30^.

Ten daggers were selected for the research, eight from area B and two from area A (Fig. 1C). The sample offers a broad range of blade morphologies, lengths, and hafting arrangements widespread in the Bronze Age including leaf-shaped and triangular blades. Except for one specimen, whose bronze handle was cast with the blade (Fig. 1C, 8), all dagger blades were riveted to handles made of now-disappeared organic materials. Chronologically, the daggers span the period from *c*.1550–1250 BCE, as revealed by their find contexts and distinctive typologies (SI Appendix, S1).

## Results

Microscopic observation and SEM–EDX analysis revealed traces of organic residues preserved on the cutting edges, blades, and hafting plates or tangs of the daggers. Using Picro-Sirius Red (PSR) solution as a staining material allowed us to identify micro-residues of collagen and associated bone, muscle, and bundle fibers of tendon, suggesting that the daggers had come into contact with multiple animal tissues. SEM observation showed the residues to be clustered along the cutting edges and at the junction between dagger blade and hafting plate/tang. The residues were mostly trapped within metal corrosion products and striations sited on cutting edges, which we interpret as use marks^10, 11^. A similar association of organic residues and striations was observed on replica daggers used for experimental butchering and the working of hard and soft animal tissues (SI Appendix, S2). The interpretation presented here is supported by SEM–EDX analysis of the residues extracted from the archaeological daggers. The analysis revealed abundant hydroxyapatite (HA), a calcium phosphate present in the mineral fraction of the bone^31^.

Previous research clarifies the conditions—such as contact between organic matter and the copper metal—that prevent bacterial decomposition while preserving animal tissue intact. Langejans^32^ demonstrates that combined salt and metals can inhibit the activity of microbes and enzymes, enabling protein-muscle tissue to survive. Likewise, Grömer^33^ argues that the acids and tannin contained in soil sediments (e.g., peat bogs) enable preservation of protein-rich organic matter (e.g., wool, skin, hair, and horn), while Janaway^34, 35^ makes a similar argument for textiles preserved within metal corrosion products.

### Organic residues

**Figure 2:**
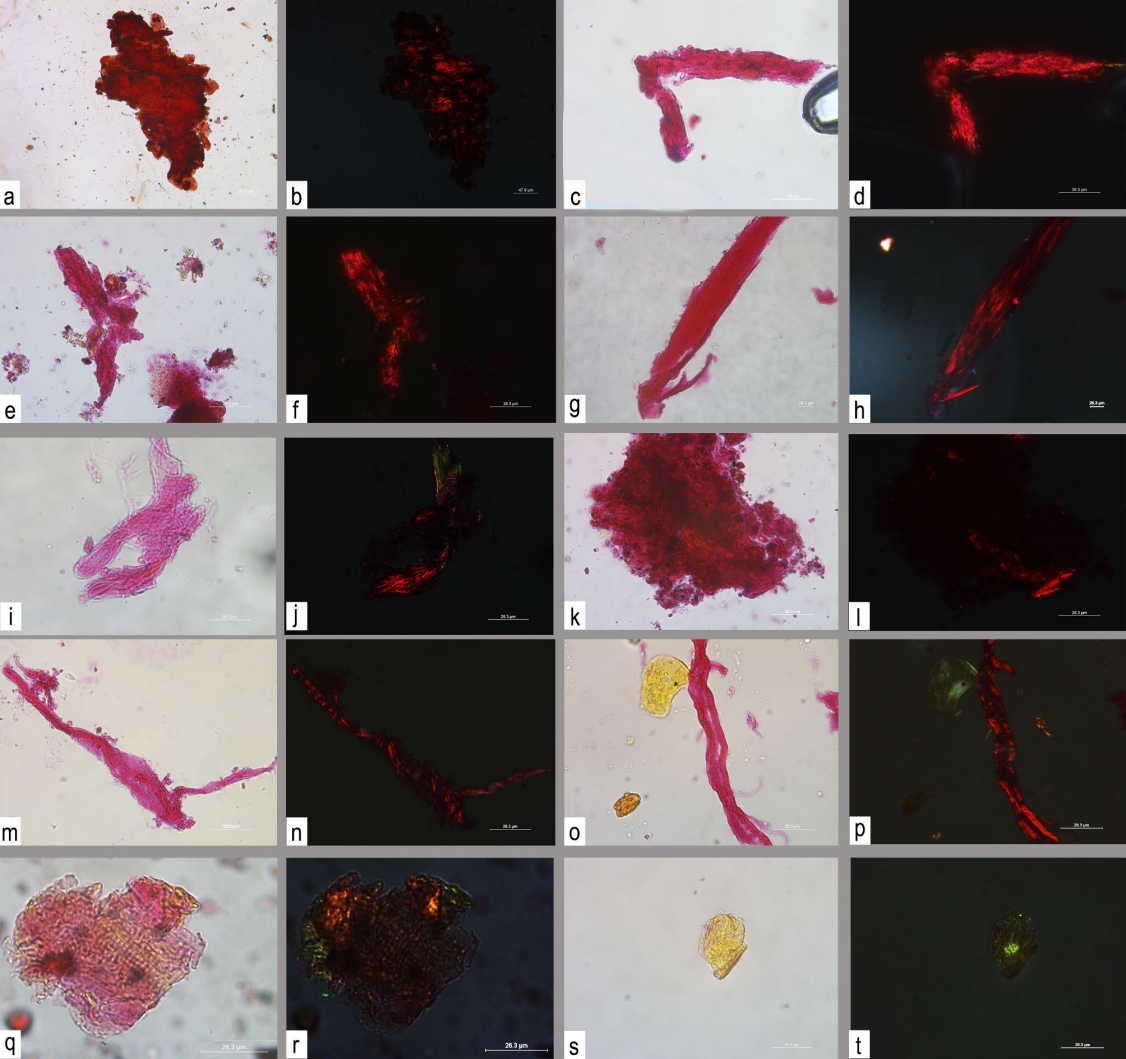
Archaeological residues observed in transmitted and cross-polarized light with staining compound PSR. (**a**,**b**) sheets collagen with an angular outline; (**b–f**) amorphous compact residues with a rough/cratered surface and peripheral crystalline fragments; (**g**,**h**) tissue with longitudinal grooves; (**i**–**p**) bundles of fiber; (**q**,**r**) striated muscle tissue; (**s**,**t**) amorphous matter.

**Figure 3:**
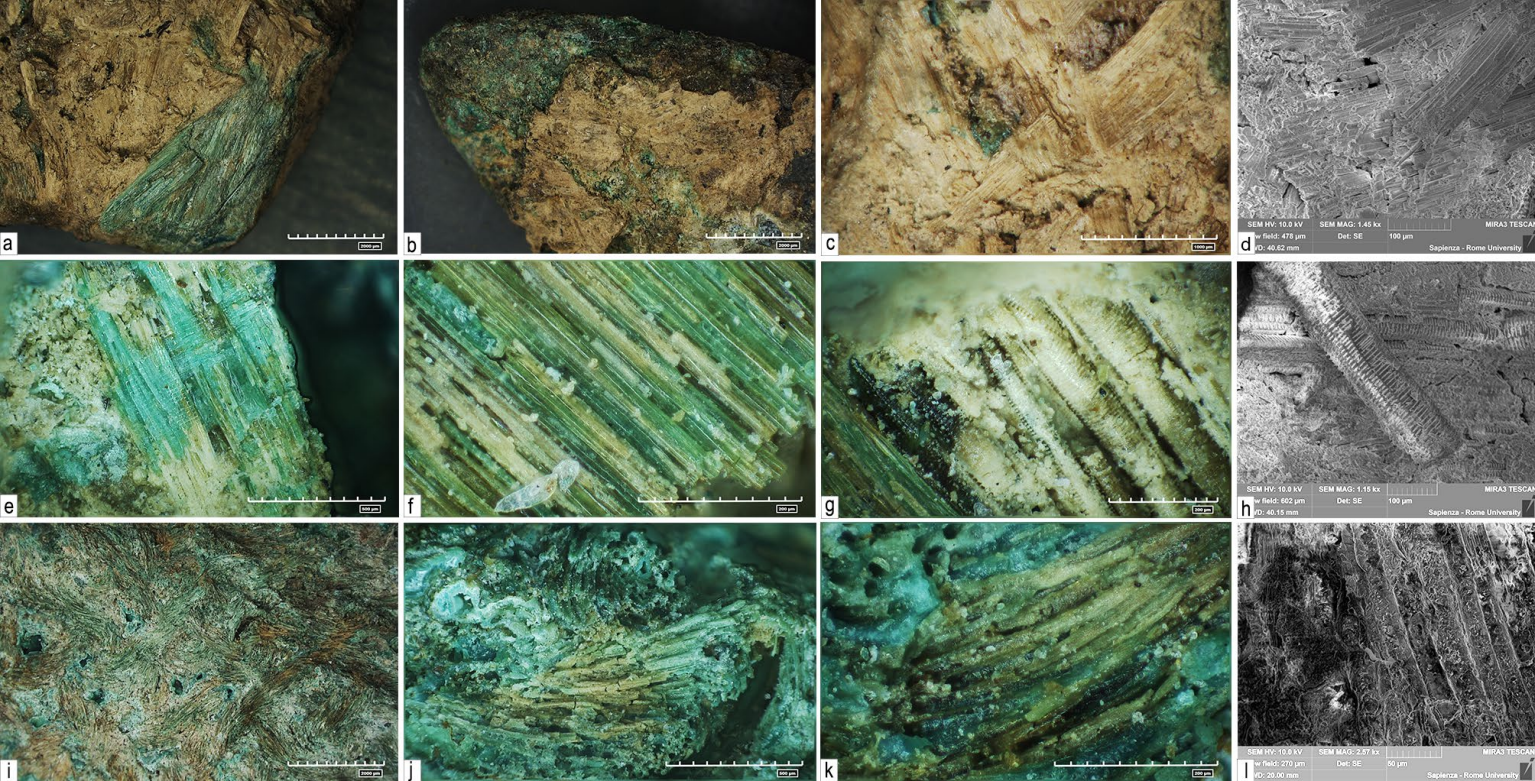
Residues observed on the copper-alloy daggers from Pragatto, interpreted as remnants of sheaths. (**a**–**h**) specimen no 2037 observed with an RH-Hirox digital microscope displays intertwined plant fibers interpreted as *cfr. Alnus*; (**h**) SEM imaging (MIRA3 by Tescan) of sample no 2037 highlights details of xylem plant cells and water conducting tissues; (**i**–**l**) specimen no 1707 observed with an RH-Hirox digital microscope displays residues of non-determined fur fibers; (**l**) details of the negative cast of the animal fur residues as observed with a SEM microscope (MIRA3 by Tescan).

Organic residues were searched for on all ten daggers. Areas observed included blade face, cutting edge, point, and hafting plate or tang (both sides). We identified organic residues on eight specimens (SI Appendix Tables S2, S3). The residues belonged to the following categories: (1) type I and III collagen^36^, mainly clustering on the cutting edges; these are interpreted as use-derived residues (Fig. 2); (2) mineralized residues of fibers belonging to (a) several species of plants, one of which appears to be *cfr. Alnus* (SI Appendix, Section S1); these are interpreted as remnants of dagger sheaths an artefact types known from the Alpine Iceman’s lime tree bast dagger sheath^37^; and (b) worked non-determined animal fur, which is also interpreted as a dagger sheath residue (Fig. 3; SI Appendix, S1); (3) bone residues clustering on the hafting plates or tangs, which are interpreted as handles or handle plates (SI Appendix, section S1); and (4) soil contamination including starch grains of Triticeae, feathers, raphides (i.e., calcium oxalate crystals), and an animal hair. Examination of a soil sample associated with one of the daggers supports our view that these are environmental contaminants, not use-derived residues (SI Appendix, section S1-Fig.S3). No collagen residues are present in the soil, similar to those found on metal blades.

Micro-residues were sampled from the eight daggers using the PSR staining procedure described below (see “Materials and methods”). The residues were mostly embedded in the metal corrosion patina coating the objects. Corrosion is a natural alteration process affecting most types of metal and alloy, which is caused by a wide variety of factors, most commonly electrochemical reactions and oxidation. Depending on taphonomic conditions (e.g., temperature, pH, and soil composition), minerals and crystals may grow on the metal itself; their structures depend on the type of organic matter they have been in contact with^38^. Dagger corrosion showed under the microscope as irregular bands or spots ranging in color from green to orange/red, associated with inter-granular attack and pit formation (SI Appendix, section S1). Similar corrosion structures developed on the replica daggers used in carcass-processing experiments (SI Appendix, section S2).

Overall, results of microscopic observation and analysis of use-derived organic residues (point 1 above) can be summarized thus: (A) three types of micro-residues are interpreted as bone, namely: (A1) sheet collagen with an angular outline, which could represent damaged periosteum, characterized by a high birefringence and appearing predominantly red in cross-polarized light, with rare yellow/orange spots (Fig. 2a,b); (A2) amorphous compact residues with a rough or cratered surface and peripheral crystalline fragments; under cross-polarized light, these residues appear red with medium–high birefringence (Fig. 2c–f); (A3) bone tissue with longitudinal grooves (Fig. 2g,h). (B) Fibers, namely (B1) bundles of fiber, dense collagen which may represent tendon, appearing red in cross-polarized light with medium–high birefringence (Fig. 2i–p); (B2) striated muscle tissue; they appear orange and green in cross-polarized light with a medium–low birefringence (Fig. 2q,r). (C) Unidentifiable amorphous residues (Fig. 2s,t). All types of residue are described in Tables (SI Appendix Tab. S2, S3).

### SEM–EDX analysis

**Figure 4:**
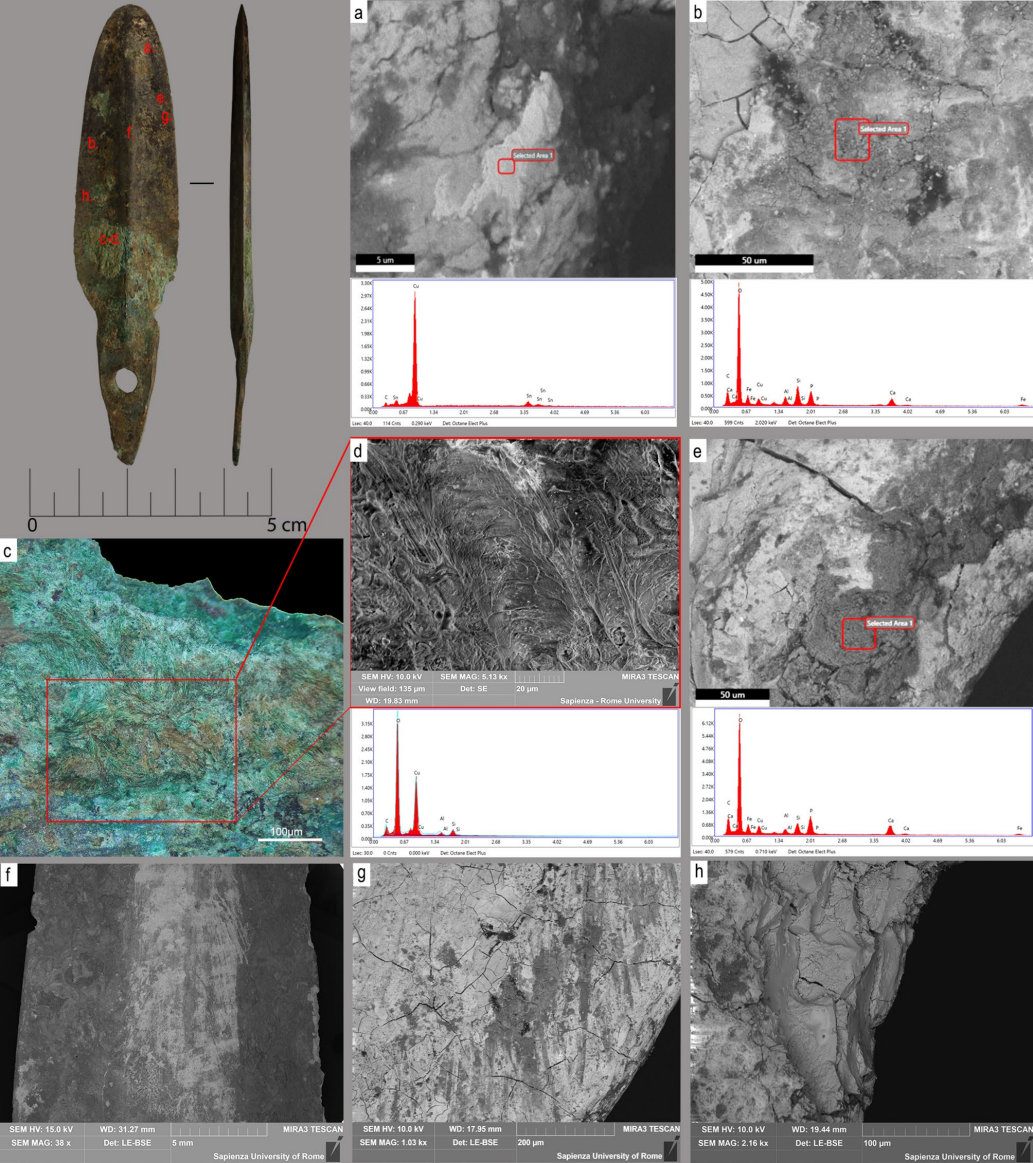
SEM–EDX analysis of dagger no 1707. (**a**) alloy composition (Cu–Sn); (**b**) analysis of corrosion structures associated with an amorphous residue (C–O–Ca–P); (**c**,**d**) analysis of mineralized fur fibers associated with a C–O–Cu compound and Si–Al; (**e**) compact amorphous material found along the cutting edge, associated with a C–O–Ca–P compound and Cu; (**f**) portion of the blade analyzed by SEM–EDX; (**g**) oriented use striations associated with organic residues as observed on the cutting edge; (**h**) corroded and flaked surface of the dagger blade.

Scanning Electron Microscopy coupled with Energy-Dispersive X-Ray analysis (SEM–EDX) was performed on five daggers from the sample. High-power observation and microprobe analysis were carried out on multiple spots (4 ≤ p ≥ 8) on both faces of the blades and hafting plates or tangs. The analysis highlighted mineralized areas rich in organic residues, as well as better preserved areas that were selected for alloy composition analysis (Fig. 4a). All specimens analyzed displayed varying amounts of Sn with traces of Fe, as is common for Middle-Late Bronze Age metals from northern Italy; no As or Pb were detected^39, 40^. Corrosion structures including cracks and flaked surfaces were observed under the SEM. As one would expect, these were concentrated along blade edges where the metal is thinnest and most susceptible to degrading (Fig. 4f–h).

Specimen no 1707 returned some of the best-preserved organic residues in the sample. SEM–EDX analysis revealed traces of fur fibers, which we interpret as remnants of a dagger sheath. The fibers were associated with Cu and a C-O-Si-Al compound likely resulting from the sheath’s mineralized fur/hair remains (Fig. 4c,d). Residues consisting of dark amorphous matter were also observed (Fig. 4e). These are located along the cutting edge and trapped within use-generated striations. Their analysis returned an association of Ca/P in a reasonable ratio (specimen no 1707, Fig. 4, Atomic% ratio Ca/P point b = 1.43; Ca/P point e = 1.35; Weight % ratio Ca/P point b = 1.85 and point e = 1.75). Specimen no 1798 yielded similar results (Atomic% = 1.33 and Weight% = 1.73). These parameters are indicative of hydroxyapatite Ca10 (PO4)6 (OH)2, a calcium phosphate present in the mineral fraction of the bone^31^.

An important feature of hydroxyapatite is its stability relative to other calcium phosphate compounds under physiological conditions including temperature, pH, and composition^41^. Published experiments show that heated bone has elemental atomic percentage values between 1.07 and 1.67 for defleshed bone and between 1.42 and 1.93 for fleshed bone. Weight percentage values of defleshed bone lie between 1.66 and 2.35, while the bracket is 1.84–2.50 for fleshed bone^41^. These values are broadly in line with those observed on specimens 1707 and 1798, as is their Ca/P atomic ratio (as determined by EDX analysis) vis-à-vis the literature^17, 22, 42, 43^. Overall, these studies support our view that the daggers from Pragatto had been in contact with bone tissue.

## Discussion

The function of prehistoric copper-alloy daggers has long been debated, with the controversy often extending to coeval flint daggers^5, 44^. Traditionally, these objects were viewed as symbolic signifiers of male identity and power—a reading predicated upon dagger-rich warrior burials^45, 46^. Not all authors agree with this view, however, some scholars suggest that daggers would be placed in warrior burials following lifetime deployments as close-range weapons^47–49^. This reading is supported by occasional skeletal injuries that may have been inflicted by daggers^50–52^. Other scholars maintain that daggers might have been multifunctional tool-weapons, or perhaps specialized tools employed in the ritual slaughtering of livestock^53–55^. The debate is largely speculative due to the scarce scientific data.

The research discussed in these pages has remedied the situation. The data emerging from integrated organic-residue and SEM–EDX analysis, validated by experiments with replica daggers, indicate that prehistoric metal daggers were used to process animal carcasses. The evidence shows interaction with both hard and soft tissues. This suggests that daggers were used for a wide range of tasks that followed (and perhaps comprised) the slaughter of farm animals and game including butchering the carcass and carving the meat from the bone (as shown by notable bone, tendon, and muscle residues). The evidence tallies with the usewear studies reviewed above, which point to a widespread desire for keeping daggers sharp throughout their use lives. It is also in line with Pragatto being a settlement site where animal husbandry was extensively practiced^23^ and our own experiments, which documented how effective these tools can be in detaching soft tissue from the bone.

Significantly, the reading proposed here is independently validated by the microwear analysis of butchered animal remains from Chalcolithic and Bronze Age sites, which frequently show metal cut marks^56–60^. Of course, daggers may have had additional uses, e.g., as close-range weapons. Further research is needed to build a comprehensive functional interpretation of early metal daggers; this is now possible thanks to the replicable methodology detailed below.

## Materials and methods

### Experiments with replica daggers

Eight bronze daggers were cast, finished, and sharpened using prehistoric technologies and methods. The replicas were based on European Chalcolithic and Bronze Age templates including blade geometries close to the specimens from Pragatto. The replicas were cast from two alloy compositions: 4% tin-bronze (n = 3), providing a compositional proxy for Early to Middle Bronze Age low-tin alloys, and 10% tin-bronze (n = 5), reflecting Middle to Late Bronze Age high-tin alloys. All replicas were subjected to a single cycle of mechanical edge hardening and were subsequently sharpened.

All daggers were utilized for cutting, scraping, and drilling activities lasting from 3 to 5 h each (SI Appendix, section S2). The experimental protocol encompassed three phases: (1) four daggers were used to process animal bone, tendons, muscles, and cartilage; their residues were then isolated on the objects and described through microscopic observation; (2) two daggers were used for butchering and carving the carcass of a pig (*Sus scrofa*) and of a red deer (*Cervus elaphus*); this helped us to document associations between residues; and (3) two daggers were used to work green and dry wood and harvest *Triticum monococcum* and *Triticum dicoccum* wheat. Seven to ten days after use, we observed oxidation structures appearing on top of the plant and animal residues, whose color ranged from orange/green to black. The residues and corrosion structures were observed and described using the microscopes discussed in “Organic residue and SEM–EDX analysis” (SI Appendix, section S2; Figs.S5-S8).

### Organic residue and SEM–EDX analysis

Ten archaeological daggers and eight experimental replica daggers were analyzed at the Wolfson Archaeology Laboratory, Newcastle University (UK), searching for macroscopic and microscopic organic residues. Macro-residues were observed using (1) a binocular stereomicroscope Leica EZ4 W with integrated 5-megapixel camera (magnification range 8×–35×); and (2) a reflected-light metallographic microscope Res Micro Leica DM2700 MH RL with integrated LEICA MC170 HD camera (magnification range 5×–50×). Micro-residues were observed under a trinocular polarizing microscope Res Micro Leica DM750P with built-in LEICA MC170 HD camera. The microscope had progressive magnifications, i.e., 5×–10×–20×–65× and a 100× immersion-oil lens. Subsequently, the archaeological daggers were observed and chemically characterized at the Department of Engineering, Sapienza University of Rome (Italy) using a field-emission SEM (MIRA3 by Tescan) equipped with an EDX detector (EDAX—Octane Elect, model Elect Plus, Sensor Area: 30 mm^2^), while the replica daggers were examined at Newcastle University (UK), Faculty Analytical SEM Unit, using a Jeol 5610LV SEM with an Oxford ‘X-act’ thin-window EDX system for elemental analysis. Prior to SEM–EDX analysis, both archaeological and replica daggers were gently washed with ultra-pure water to remove any adhering sediment. The objects were not treated or conserved until after completion of the analysis so as not to jeopardize data collection and interpretation.

### Sampling and description of macro- and micro-residues

Macro-residue description followed the procedures discussed by Fullagar and Matheson^61^; Hayes and Rots^17^; and Lombard^62^. Variables of interest encompass localization, color, morphology or structure, appearance, and birefringence. Following Stephenson^36^, we developed a micro-residue sampling and observation protocol using the biochemical staining Picro-Sirius Red Solution (PSR) (ab246832-Abcam^®^). This is a strongly acidic azo dye used in histology to stain biological tissues and collagen surviving in millennia-old archaeological contexts^15, 63^. Importantly, PSR is proven not to alter the morphology and structure of plant residues such as starch grains and phytoliths^64–66^. Once treated with PSR and observed under cross-polarized light, Type I collagen appears as thick, strongly birefringent red or yellow fibers; Type II collagen displays a weak birefringence of varying color; and Type III collagen appears as thin, weakly birefringent green fibers^36, 67^.

Samples were taken from 10 ≤ p ≥ 15 points on the dagger surfaces on both sides of the blades focusing on tips, cutting edges, and hafting plates or tangs. The samples were taken from oxidized structures as well as other spots where low-power microscopy had revealed details of interest. Both archaeological and experimental daggers were sampled. Two methods of micro-residue sampling were tested, namely (1) wet sampling using a pipette and mineralized water, and (2) dry sampling. We found dry sampling to provide higher amounts of residue and thus to be preferable to wet sampling. Dry sampling is also advantageous for long-term sample storage since it does not wet or alter the metal specimen. We extracted the dry samples using a sterile probe or scalpel (in the latter case using the blunt edge of the tool to avoid scratching the metal). We found it sufficient to take 0.5–1 mg of corroded metal and place it in a sterile microcentrifuge tube.

The next step of the analysis took place in a sterile laboratory environment. The dry sample was placed in a sterilized agate mortar and finely pulverized, adding 3–4 drops of ultra-pure water with a Pasteur glass pipette. The pulverized sample was then placed on a clear slide using a micro-pipette. As two drops are normally sufficient for the analysis, the same sample may be used for several slides. The slide was left open (i.e., without glass cover) in a petri dish and allowed to dry in a biosafety cabinet to limit contamination. Subsequently, the PSR stain was applied following the protocol described by Stephenson^36^. The tissue was completely covered with PSR solution and left to incubate for 60 min. The slide was then rapidly rinsed with Acetic Acid Solution (0.5%). Finally, a mounting media (50% glycerol and 50% ultra-pure water) was placed on the stained area and covered with a coverslip. The coverslip was not sealed to allow subsequent rehydration of the sample, as needed. The sample was then ready to be observed in both transmitted and cross-polarized light.

## Supplementary Information

Supplementary Information.

## Data Availability

All data generated or analysed during this study are included in this published article [and its supplementary information files].
