# Supplementary material for: Organic residue analysis reveals the function of bronze age metal daggers
Source: Sci Rep. 2022 Apr 12;12:6101. doi: 10.1038/s41598-022-09983-3 (PMC9005664; doi:10.1038/s41598-022-09983-3)
Supplement: Supplementary file 1 — Supplementary Information. [file 41598_2022_9983_MOESM1_ESM.docx]

**Supplementary Information for:**

**Organic Residue Analysis Reveal the Function of Bronze Age Metal Daggers**

**Isabella Caricola^*^, Alasdair Charles, Jacopo Tirillò, Fraser Charlton, Huw Barton, Francesco Breglia, Alberto Rossi, Maria Chiara Deflorian, Anna Maria De Marinis, Susanna Harris, Alessio Pellegrini, Federico Scacchetti, Paolo Boccuccia, Monica Miari, Andrea Dolfini***

**Corresponding authors:**

*[isabellacaricola@gmail.com](mailto:isabellacaricola@gmail.com) Newcastle University, School of History, Classics and Archaeology, Newcastle Upon Tyne (UK), Armstrong Building, NE1 7RU, telephone: +39 3284623364; ORCID: 0000-0001-7808-3748

*[andrea.dolfini@newcastle.ac.uk](mailto:andrea.dolfini@newcastle.ac.uk) Newcastle University, School of History, Classics and Archaeology, Newcastle Upon Tyne (UK), Armstrong Building, NE1 7RU, telephone: +44 (0)191 2083402.

**This PDF file includes:**

Supplementary text

Figs. S1 to S10

Tables S1 to S7

SI References

SUPPLEMENTARY INFORMATION

Supplementary text

Section S1. Supplementary archaeological data……………...……..................................................3

Section S2. Experimental trials…......………………….....................................................................6

Figures S1 to S10 ...............................................................................................................................9

Tables S1 to S7 .................................................................................................................................19

References .........................................................................................................................................26

**Section S1. Supplementary archaeological data**

**The site**

The *Terramara* of Pragatto lies in the Po River Valley (Northern Italy), along the Apennine foothills between the modern cities of Modena and Bologna. The area was already known in the past for the discovery of a cemetery bearing the same name and a small village dated to the Middle Bronze Age, horizon 2^1,2^. New excavations carried out in 2016-2017 brought to light a larger village, whose stratigraphy and material culture highlighted three settlement phases spanning the Middle Bronze Age, horizons 2-3, and the initial Late Bronze Age, c.1550-1250 BCE overall^2^.

The excavation was operationally divided into three areas named A, B, and C. The topmost layers of the archaeological deposit were damaged by plowing in most places. However, the stratigraphy was better preserved in the southern portion of Area B. This allowed recognition of the following stratigraphic sequence (bottom to top): *a*) conical drains of gray and reddish ash were at the base; *b*) levels of compact yellow silt, probably the result of the decomposition of the earthy structural elements (i.e., floors and walls); *c*) layers rich in organic material, with fragments of adobe and heterogeneous archaeological materials. Areas A and B mainly featured houses and minor structures interpretable as storage rooms. All houses lay very close to one another. They were seemingly built with the wooden deck technique common at most *Terramara* sites, as suggested by the discovery of post holes and timber board remains. Area B alone yielded nine houses belonging to a single phase of the prehistoric village (**Fig. S1.**). The excavators identified a complex perimeter delimitation system, including a moat, palisade, and wooden gabion^2^. These structures are comparable with those previously discovered at the *Terramara* of Castione dei Marchesi (Parma, Italy)^3,4,5^.

The site did not yield metalworking features. However, two molds and fragmented bronze items suggest *in-situ* metal casting and processing, which is commonplace at *Terramara* sites^6^. The site returned over 150 copper-alloy objects: metalwork encompassed daggers, arrowheads, dress pins, and numerous craft tools of various descriptions. The site also yielded abundant fauna, ceramics, stone tools, and objects made of animal bone and horns. Moreover, several timber structures (e.g., gabions) were found. Their preservation is because of the presence of iron salts in the soil and the burning episodes that repeatedly affected the site in the Bronze Age^2^.

**Additional data on archaeological metal daggers**

Various morphologies characterize the analyzed archaeological bronze daggers from Pragatto. These include daggers with foliate or triangular blades, in some cases with a central rib. The daggers may present 1 or 3 rivets and different bases (e.g., simple base or tang). The dimensions of samples range between 60 mm and 170 mm. Typological observations refer them to morphologies widespread in northern Italy and the Po Valley during the Middle and Late Bronze Age (e.g., “Toscanella,” “Campegine,” “Castelluccia,” “Redù,” and “S. Ambrogio” types)^7^ (**Table S1.**).

However, the daggers exhibit different corrosion degrees that appear macroscopically pervasive on all the dagger-blade surfaces. In particular, some characteristic red-orange and yellow-green corrosions are very interesting. These are more evidently located along the cutting edges and the most proximal part of the handle. These corrosions can appear in a form of irregular bands or spots; in some cases, they can be a few millimeters thick (1mm) (**Fig. S2.**). Despite the corrosions, different portions of the blades are perfectly preserved, and it is possible to observe striations connected both to sharpening and use^8^. It was experimentally demonstrated that the traces found on the daggers correspond to specific activities. One can distinguish those caused by production from those caused by the use^9,10^. To identify the usewear traces, some replica daggers were crafted and tested on various materials such as wood, reeds, leather, and meat, performing tasks such as carving, whittling, chopping, slicing, scraping, and stabbing. Comparing replicas and archaeological daggers allowed us to find analogies and differences in the traces. Most notably, we identified hammer marks caused by the work hardening of the blade, especially on sample 264. Most striations were interpreted as grinding traces, considering their longitudinal direction and position on the blade. Sharpening traces were found on the edges of many samples, featuring short, diagonal, and densely packed striations that overlap the hammer marks and the polishing traces. However, many traces were interpreted as use-wear considering their patterning and direction. Some edge damage appeared to be caused by the interaction with hard matters, such as bone. Activities such as scraping or cutting generated almost horizontal isolated striations, with a substantial difference in depth, packing and appearance depending on the worked material. Many experimental traces matched perfectly those observed on the Pragatto daggers: mainly indentations, bending, and striations^8^.

**Residues of Sheaths**

Residues composed of animal fur/hair and plant/wood was identified on four daggers. More specifically, fur residues were observed on one sample (i.e., n.1707). It is impossible to determine the species because of the partiality of the conservation of the fur. Many hairs were preserved by the negative cast and metal corrosion product. In this latter case, the hairs appear three-dimensional and mineralized.

Likewise, the intertwining of plant material was identified on three daggers (i.e., n.2037; n.175; n.1321). The most conserved enabled extracting some samples to be subjected to detailed botanical analyzes (i.e., n.2037). At the metallographic microscope, n. 5 fragments of wood tissue were observed on the dagger n. 2037. The samples are pertinent to a single artifact made of organic material, preserved, by mineral replacement, only in its portion adhering to the metal blade of the dagger. Of the n. 5 fragments analyzed, two samples are 1-1.5mm in length/width and 0.2 mm in thickness; the other three were not useful for the determination. One sample presents a black color compatible with burnt areas, also macroscopically visible on the dagger.

In all fragments analyzed, typical anatomical structures of the secondary xylem of dicotyledonous angiosperms were observed, in this case, fibers, vessels, and rays. This confirms the relevance of the analyzed wood tissue to broadleaves.

It was possible to observe only the radial section in four cases because the other sections (transverse and tangential) were unreadable. The impossibility of observing the cross-section makes it very difficult to identify the relevant species. However, the black fragment presents a tangential section characterized by homogeneous uniseriate rays, between 5 and 20 cells high, rarely bi-triseriate, aggregated rays, and scalariform perforations. These characteristics are typical of *Alnus sp.* (i.e., Alder).

During the observation of radial sections from the other fragments, no scalariform perforations were observed. Spiral thickenings were instead identified in the fibers (not present in *Alnus sp*.); the vessels are grouped in clusters and have a reticulated wall. Finally, the rays sometimes exhibit one or two rows of marginal square cells. These elements are not sufficient to determine the relevant species. However, they allow us to define that at least two different types of wood were certainly used.

The use of different types of wood seems to be further confirmed by identifying some isolated fiber-tracheids. These presented a uniseriate row of areolate pits that would refer to a gymnosperm (i.e., conifers).

Finally, according to the different orientations of anatomical elements, we hypothesize that the artifact consisted of small thin strips of wood, detached along the radial and tangential sections of young branches, probably fresh, of different species. For this purpose, it looks appropriate to use *Alnus sp*., whose wood connotes for a straight arrangement of fibers and a fine and uniform texture and for being particularly smooth and free of knots or imperfections and for its easy workability.

These types of residues, both wood and fur/hair, identified on four daggers, were interpreted as sheaths elements.

**Contaminations**

Some residues found on the daggers mentioned in the main text require additional information. More precisely, some residues identified on the daggers can be interpreted as contaminations. Their identification is important from a methodological point of view. These residues can provide information about the type of environment and resources that characterized the Terramara during the Bronze Age. The contaminations observed are the following:

**1**) an animal hair (**Fig.S3.**), visible under the stereomicroscope on sample 1798, appears white with a milky appearance. The sample consists of a guard hair, probably referable to the GH0 or GH1 type, complete from the bulb to the tip, slightly curved, not wavy, and c. 2.2 mm long. Based on the SEM images, it is possible to hypothesize that the cross-section of the hair is approximately concave-convex. In the proximal portion, the cuticle appears to be composed of transversal scales with distant and smooth margins, arranged similarly to the regular wave pattern. The scales maintain the transverse pattern in the distal portion, with closer, slightly wavy margins. It is not clear if the scale margins can be frilled.

Finally, based on the comparison of the observable features with a reference collections housed at the MUSE (Museo delle Scienze, Trento, Italy) and the literature data^11,12,13,14^, it was possible to identify the order of Rodentia and select a group of families within it (i.e., Cricetidae, Muridae or Sciuridae). Since the medullary pattern was not recognizable, it was not possible to identify the hair at the species level.

**2**) some micro-residues, interpreted as soil contamination, were found on the daggers. These consist of starch granules, raphides (calcium oxalate crystals), sclerenchyma tissue, vegetal fibers, and feathers (**Fig. S4.**). The Picro-Sirius Red stain solution used to highlight the collagen did not alter plant residues’ preservation. More specifically, the starch grains were identified on a morphological and dimensional range basis, using the comparison collection present at the University of Leicester. The starch grains identified on the daggers are the following:

**I**) sample 2037 yielded: ***a***) two undeterminable damaged/split granules (27.3 μm); in cross-polarized light, the granules are flat and weakly birefringent; centric cross and lamellae are not visible (**Fig. S4., k-n**); ***b***) two starch grains, type A and B of probably Triticeae, with a rounded shape, centric hilum and centric cross in polarized light; size 8.2 μm and 22.1 μm; lamellae and fissures are not identifiable (**Fig. S4., i-j**).

**II**) on sample 2035, the following is present: ***a***) group I, 29 starch grains, type A and B, with an ovate shape, eccentric hilum, ranging in size between 2.6 μm and 18.6 μm in maximum dimensions with a mean size of 9.8 μm; lamellae and fissures are not identifiable. In cross-polarized light, they were weakly birefringent with a poorly defined eccentric cross (**Fig. S4., e-f**). The sample is clustered together, and it was very difficult to observe other specific features; the smaller A-types are comparable with Einkorn (*T. monoccocum*), but we cannot exclude other types of attributions; ***b***) group II, 152 round starch grains of Triticeae, types A and B, with ranging in size between 2.9 μm and 66.3 μm in maximum dimensions with a mean size of 13.6μm. These appear with a centric hilum; lamellae and fissures are not identifiable. In cross-polarized light, they are weakly birefringent with a poorly defined centric cross (**Fig. S4., a-d**).

Finally, starch granules of Triticeae were also found in a soil sampling. More precisely, 67 sub-round granules were found, types A and B, ranging in size between 2.6 μm and 47.9 μm in maximum dimensions with a mean size of 11.7 μm. The type A granules are large with a centric hilum and extinction cross, visible in cross-polarized light. These A-types do not have very well-defined lamellae; only a few sporadic cases are exceptions (**Fig. S4., q-t**).

After being measured, the starch grains dimensional data were analyzed using the free software PAST (version 4.01)^15^ to calculate the dimensional ranges, means, and standard deviations (**Table S7.**).

**Section S2. Experiments with replica daggers**

To compare the organic residues found on the archaeological samples, we developed experimental protocols. Production and use of metal daggers replicas were tested too. The experimental residues were observed, sampled, and documented using the low and high-magnification approach (i.e., stereomicroscope, metallographic microscope in transmitted and reflected light, and Scanning Electron Microscope-EDX). More precisely, the experimental micro-residues were highlighted with the addition of the Picro-Sirius Red Solution (ab246832©), using the same procedure and equipment explained in the main text (see Methods 5.0) to observe how these appear in transmitted and in cross-polarized light.

**The production and use of experimental daggers**

The replicas of eight bronze daggers were produced using a specific experimental protocol that allowed limiting the variables. More precisely, the replicas were produced using two different percentages of tin-bronze (i.e., 4% and 10%) to test two different hardness of the metals. The production phases ranged from the casting to the hammering of the cutting edges (**Fig. S5., a-c**), until the abrasion/polishing of the dagger’s surfaces using stone (i.e., sandstone and limestone) slabs of different granulometry (**Fig. S5., d-e**). Water and sand were also added for polishing. Daggers characterize the experimental collection with triangular blades of variable length (from c.70 mm to c.180 mm). The handles were realized both in deer antler and wood, in some cases with two or three rivets (**Fig. S5., h**).

The experimental daggers are used in the treatment of organic material of animal (i.e., skeletal muscles, bone, tendons, leather, cartilage) and plant origin (i.e., wood and cereals) (**Table S4.**, **Fig. S5., i-l**). To observe the combination of residues, animal tissues were processed through single contact (it is useful to isolate the different tissues accurately) and also through multiple contacts. A month after being used, we observed the manifestation of localized corrosions of orange-red color to green shades and black spots (**Figs. S6., S7., q-r**), which appeared in correspondence with the organic residues on the blade and the most proximal part of the handle. The replicas were used for up to 5 h. Only in one case, the dagger required a re-sharpening.

**Experimental results**

The macro-residues documented on the experimental replicas are of both animal (i.e., bone, fat, hairs, skeletal muscle, and leather fibers) and vegetal origin (i.e., starch grains and wood fibers) (**Table S5.**). The residues observed under the stereomicroscope and metallographic microscope in reflected light are distributed along the edge of use; they can also be found along the innermost ribs of the daggers. This factor may depend on the hardness of the processed material and, therefore, on the blade’s capacity to penetrate it. The distribution of residues can also be influenced by the action performed – i.e., cutting, scraping, or drilling. Based on the gesture, the residue may appear with a distribution parallel to the edge of use (e.g., longitudinal or transversal cut) (**Fig. S6., a-d**), rather than with a distribution perpendicular to the cutting edge (e.g., scraping) (**Fig. S6., b-c**). Furthermore, many residues tend to accumulate/deposit during use (i.e., scraping and cutting) near the junction of the handle, and inside the techno-functional striations characterizing the surface of the metal tools (**Fig. S6., e-f**).

The animal macro-residues observed using the low power-approach consist of bone, which appears white in color with a “bubble” structure, also associated with small collagen fibers (**Fig. S7., a-c**); the fat is an amorphous reddish matter (**Fig. S7., d**); hairs (**Fig. S7., e)** The skeletal muscle is characterized by a parallel reticulum of fibers (**Fig. S7., f**) and the leather, macroscopically appears amorphous and associated with hairs with different dimensions (**Fig. S7., g**). The butchering experiment returned all these organic residues associated. The cereal harvesting determined the presence of opaque crusts also associated with powder deposits (**Fig. S7., j-k**). The plant macro-residues of wood appear as a dense glossy mass of white and orange wood tissue. In some cases, cellulose fibers are recognizable (**Fig. S7., l-p**).

The high-magnification approach allowed us to determine more accurately the type and morphological characteristics of the observed organic residues. Indeed, it was possible to recognize animal residues, such as 1) tendons, parallel elongated bundles of cylindrical-shaped fibers, 1-2 µm in diameter (**Fig. S8., a-d**); 2) the damage bone can show in various combinations; that is, the periosteum appears as bundles of fibers or single flat fibers attached to the compact bone with longitudinal fissures. The fiber diameter ranges between 1 µm to 7 µm (**Fig. S8., e-j**). In addition, the amorphous compact bone residues with a rough/cratered surface and bone with a longitudinal view, overlapping fragments, angulated edges are present (**Fig. S8., k-n**). 3) Skeletal muscles appear as a parallel reticulum of fibers and cross striations (**Fig. S8., o-p**); 4) the collagen bundles in leather can appear as amorphous and wavy bundles of fibers (c.1-2 µm in diameter) (**Fig. S8., q-t**); 5) fragments of hyaline cartilage containing chondrocytes (**Fig. S8, u-v**); 6) Amorphous collagen (**Fig. S8., w-x**); 7) hairs with cylindrical structure; the presence of medullae and sometimes the scaly cuticle or outer lay. For each animal tissue highlighted with Picro-Sirius Red, we described how these appear in color in transmitted and cross-polarized light and degree of birefringence (**Table S6.**).

Finally, wood fibers (**Fig. S9.**) and starch grains were identified on the replicas used for the processing of plant material. In addition, in butchering experiments, starch grains contaminations are also documented (**Fig. S9., a**).

**SEM-EDX analysis on experimental replicas**

The experimental replicas were analyzed at Newcastle University (UK), Faculty Analytical SEM Unit, using a Jeol 5610LV SEM with an Oxford ‘X-act’, thin-window, EDX system for elemental analysis.

Two experimental bronze daggers were used in **1**) butchering activity and **2**) cutting wood. They were optically examined and areas of interest were cut out for further examination by SEM. The specimens were selected where greenish and reddish surface discoloration was seen, and other areas where damage to the blade edges was significant. For control purposes, clean areas near the hilt of the daggers were also examined.

Butcher-related residues (i.e., contact with skin, skeletal muscles, tendons, cartilage, and bone) appear as amorphous matter, sometimes with a crusty or small flake-like appearance. The EDX elemental analyzes of these residues at four points returned an average elemental composition calculated as the Atomic Weight %, specifically: C (71%), O (19.7%), and N (6.7%), which highlighted the presence of organic materials, bearing in mind that the hydrogen component cannot be detected. Small amounts of P (0.2%), S (0.2%), K (0.2%) and Si (0.6%) were also detected and traces (below 0.1% per element) of Ca, Fe, Mg, Cl. A trace of Na (also 0.1%) appeared in one case only and may be because of the accidental handling of the sample (**Fig. S10., a-d**). The proportions of Ca/P are small and do not allow us to hypothesize with any confidence on the presence of hydroxyapatite. Moreover, it is not excluded that the Na, K, Cl, and Si peaks may be attributable to contaminations, as evidenced by the finding on the dagger of starch grains and vegetable residues not pertinent to butchering. Similar types of contamination and chemical elements were observed on stone tool replicas examined by Martín-Viveros and Ollé (2020)^16^.

Wood residues appear as amorphous matter or as amorphous plant tissues. EDX analysis of six points returned an association of elements, such as C (57.4%) and O (42.6%), connected to organic matter. Peaks for Ca (0.7%) and K (0.8%), Cl (0.7%), Si (1.0%), Al (0.6%), and Mg (0.2%) were also observed at some points. There are also minor elements such as P (0.2%), S (0.1%), and Fe (0.1%). In the literature, calcium, phosphorus, and iron could be due to undetected moisture and to the soil fertility where the plant is grown^17,18^ (**Fig. S10., e-h**).


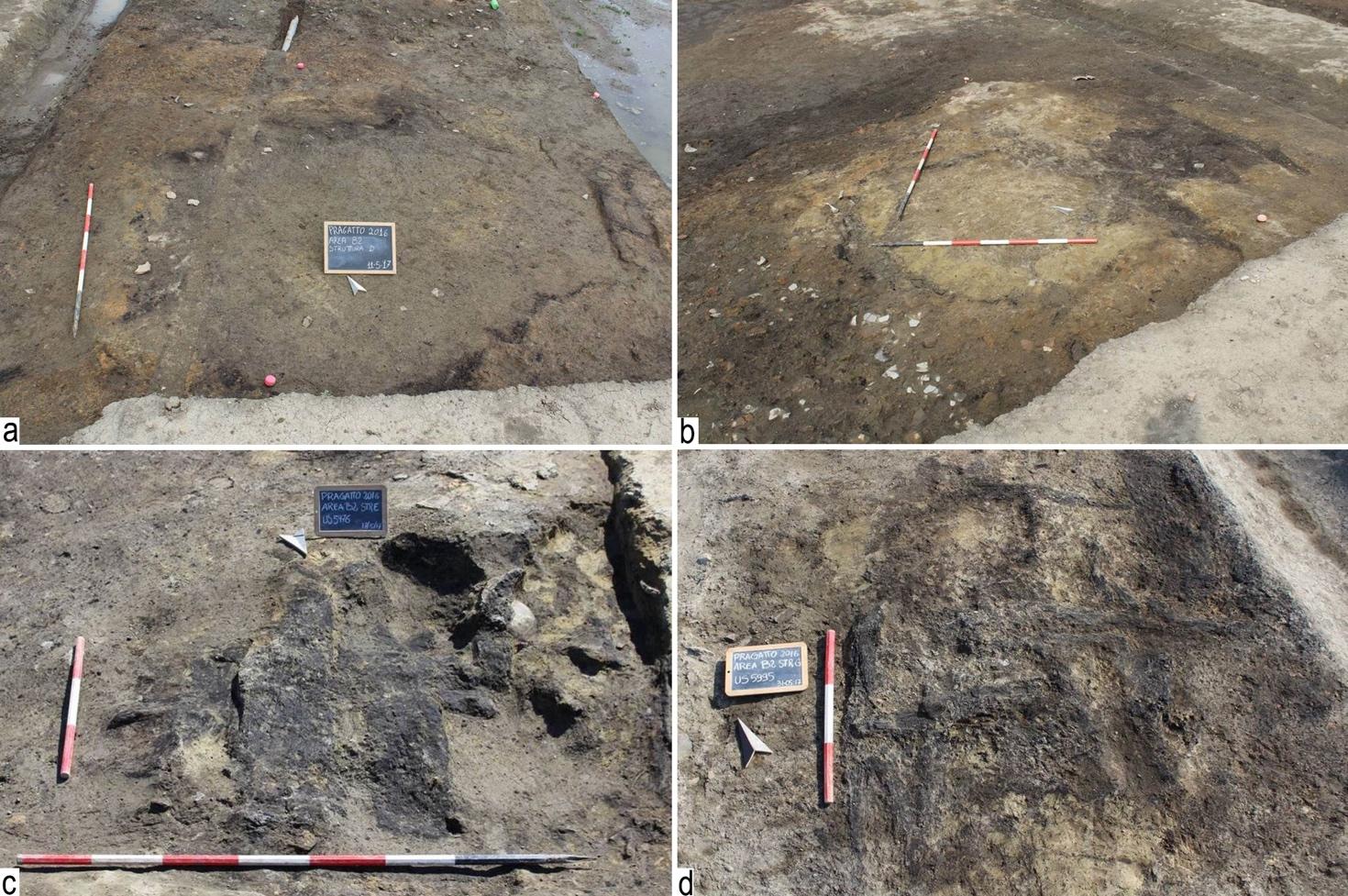


**Figure S1.** The Terramara of Pragatto (Italy). **a-d**) Some details of the burned structures found in area B.


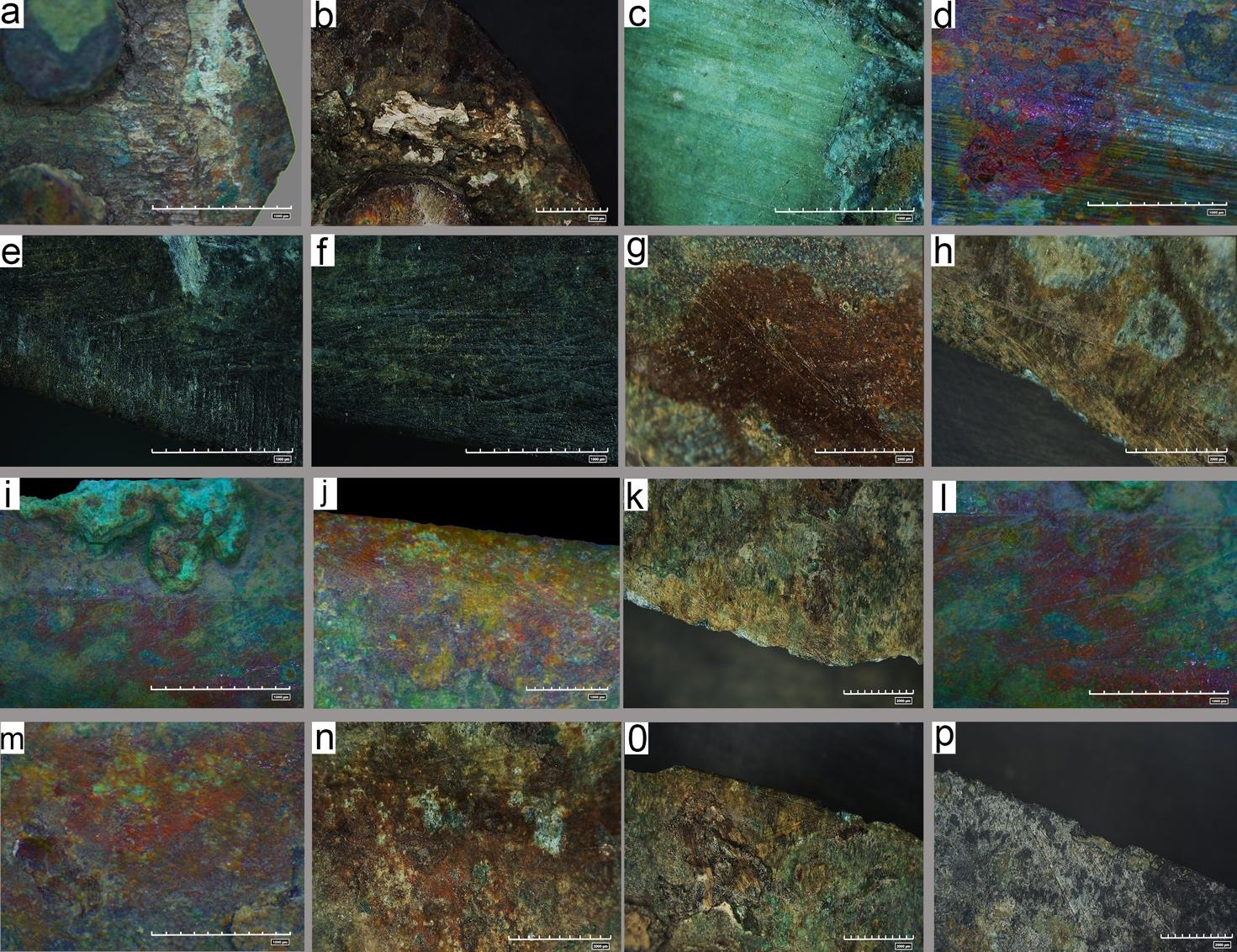


**Figure S2. Details of surfaces of archaeological metal daggers from Pragatto. a-f**) sample 264, (a,b,c) detail of the residual handle in hard animal material; (d) corrosion; (e-f) cutting edge with striations; **g-k**) sample 2035, details of corrosions; **l-o**) sample 1798, corrosion and striations; **p**) sample 1707, cutting edge and striations.


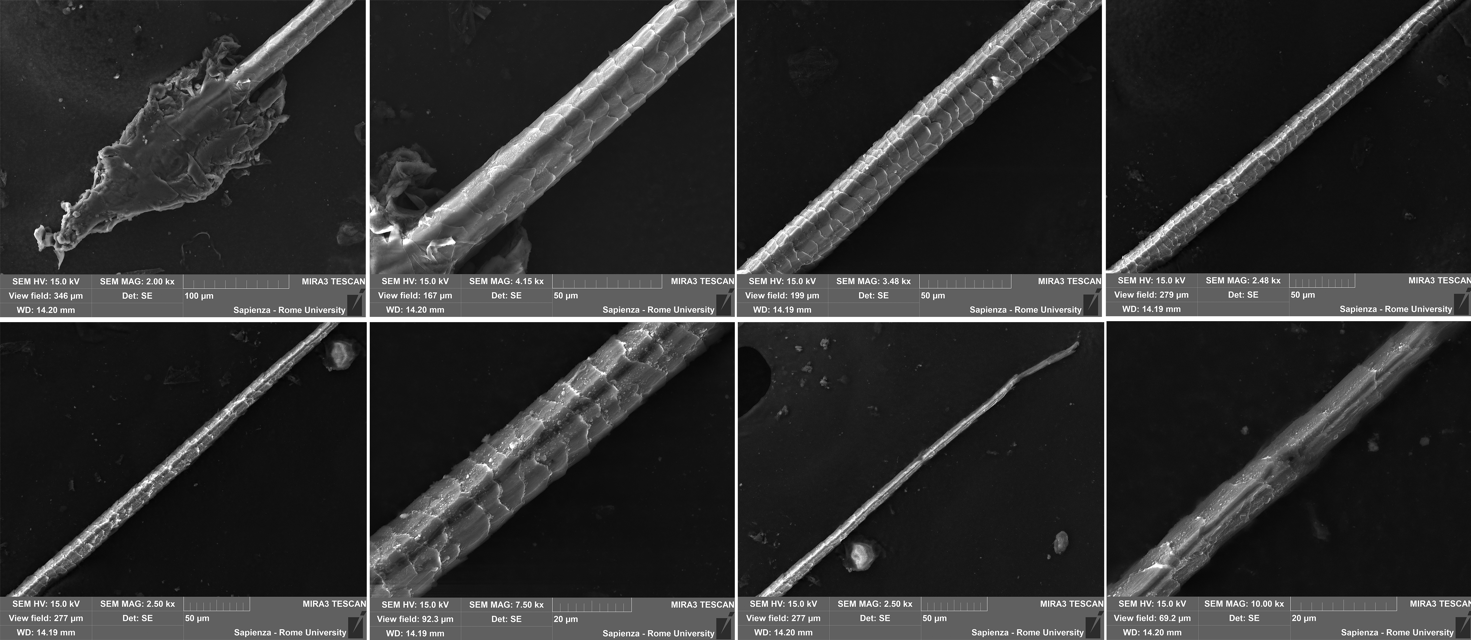


**Figure S3.** SEM image of an hair of Rodentia found on the archaeological dagger (sample 1798), interpreted as a contamination.


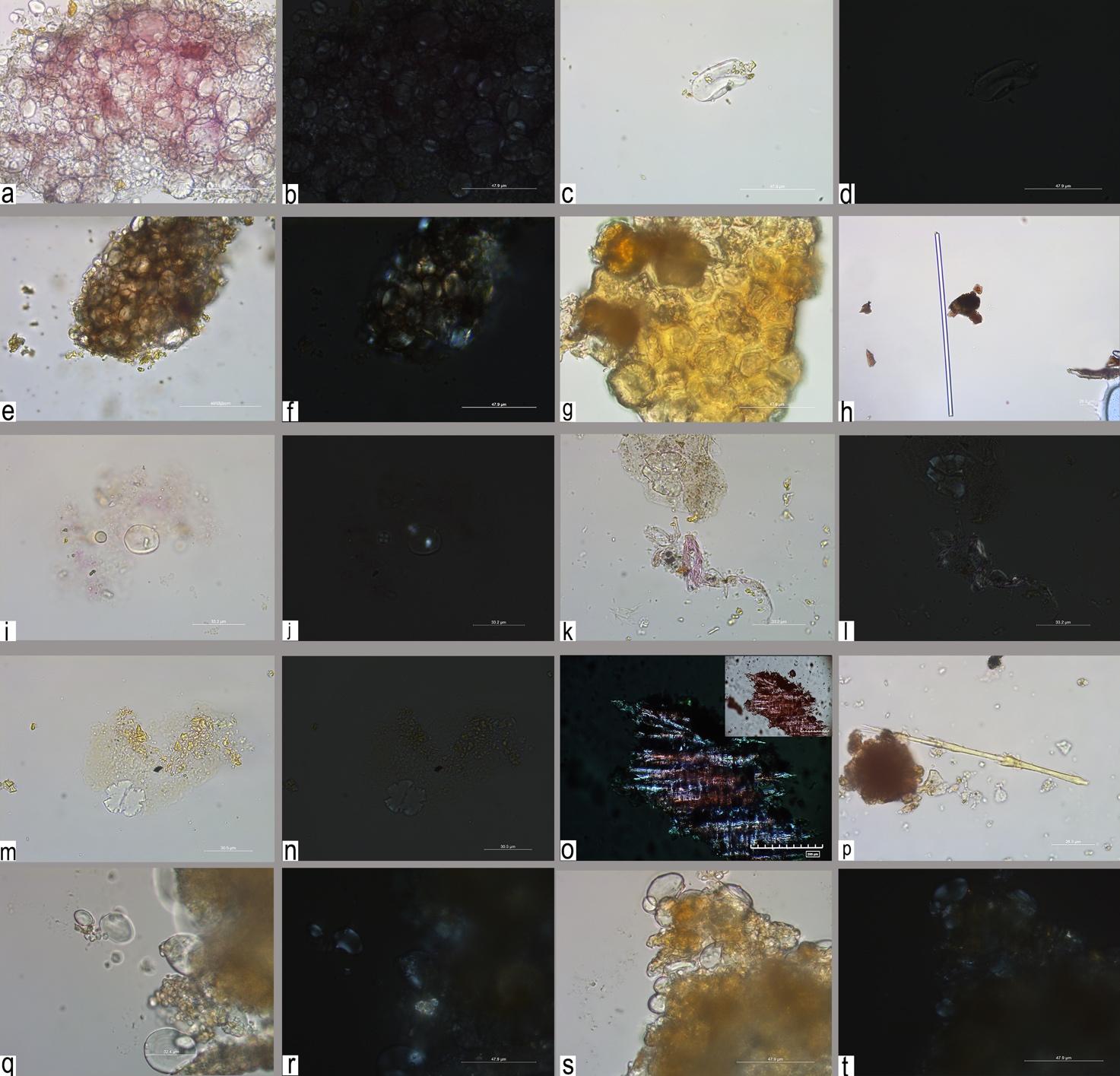


**Figure S4. Plant residues observed on the archaeological sample. a-h**) sample 2035: (a-b) group I, type A-B, starch granules; (c-d) starch grain; (e-f) group II, type A-B, starch granules; (g) thick-walled, probably sclerenchyma tissue; (h) raphide (calcium oxalate crystal); **i-p**) sample 2037: (i-j) starch grains type A and B; (k-n) damage starch grains; (o) vegetal fibers; (p) feather; **q-t**) starch grains, type A-B, from the soil sample.


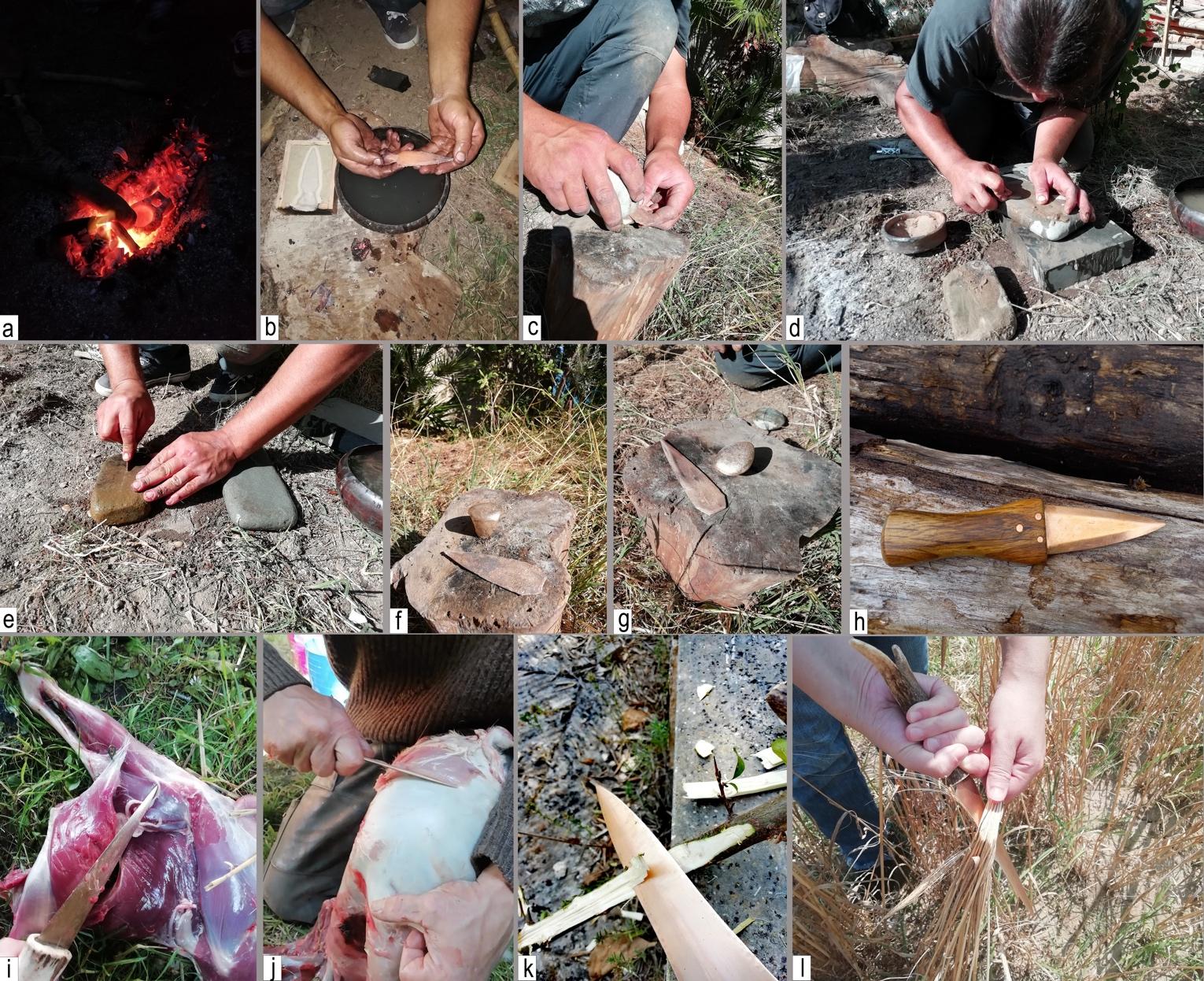


**Figure S5. Experimental protocol, production and use of bronze daggers.** **a-h**) some stages of production of the replica, in detail: (a-b) smelting; (c) hammering of cutting edges; (d-g) abrasion and polishing of the dagger’s surface; (h) the finished dagger; **i-l**) some examples of experimental use of daggers, in detail: (i) butchering; (j) leather scraping; (k) woodworking; (l) cereal harvesting.


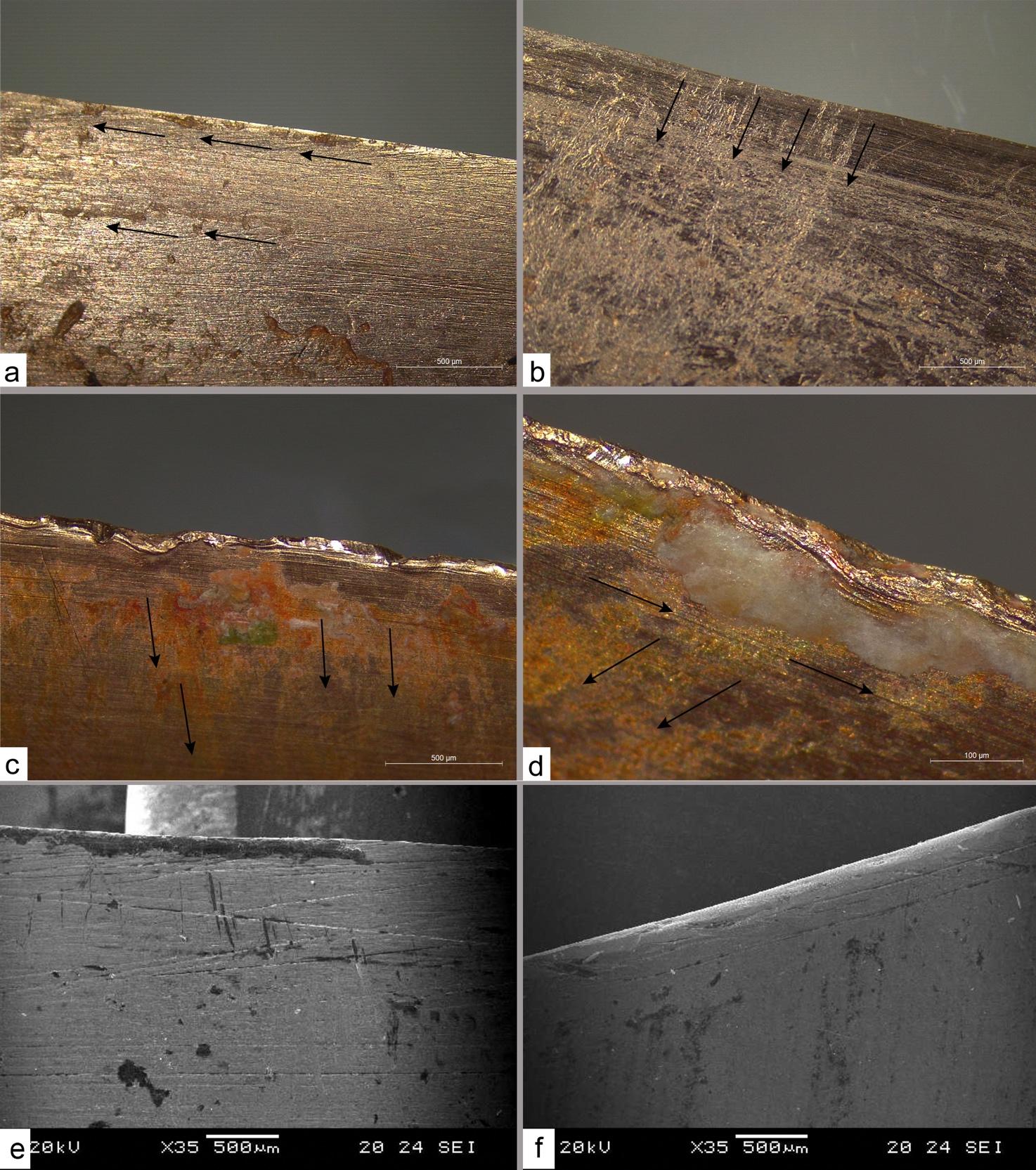


**Figure S6.** Highlighting of the distribution of the residue based on the gesture performed during the experiments. **a**) unidirectional longitudinal cut of the muscle tissues; **b**) leather scraping; **c**) wood scraping; **d**) longitudinal and transversal wood cutting; **e**) SEM image of organic residues connected to the longitudinal cutting of animal tissues, in part entrapped inside the technological and functional striations; **f**) SEM image of wood scraping residues.


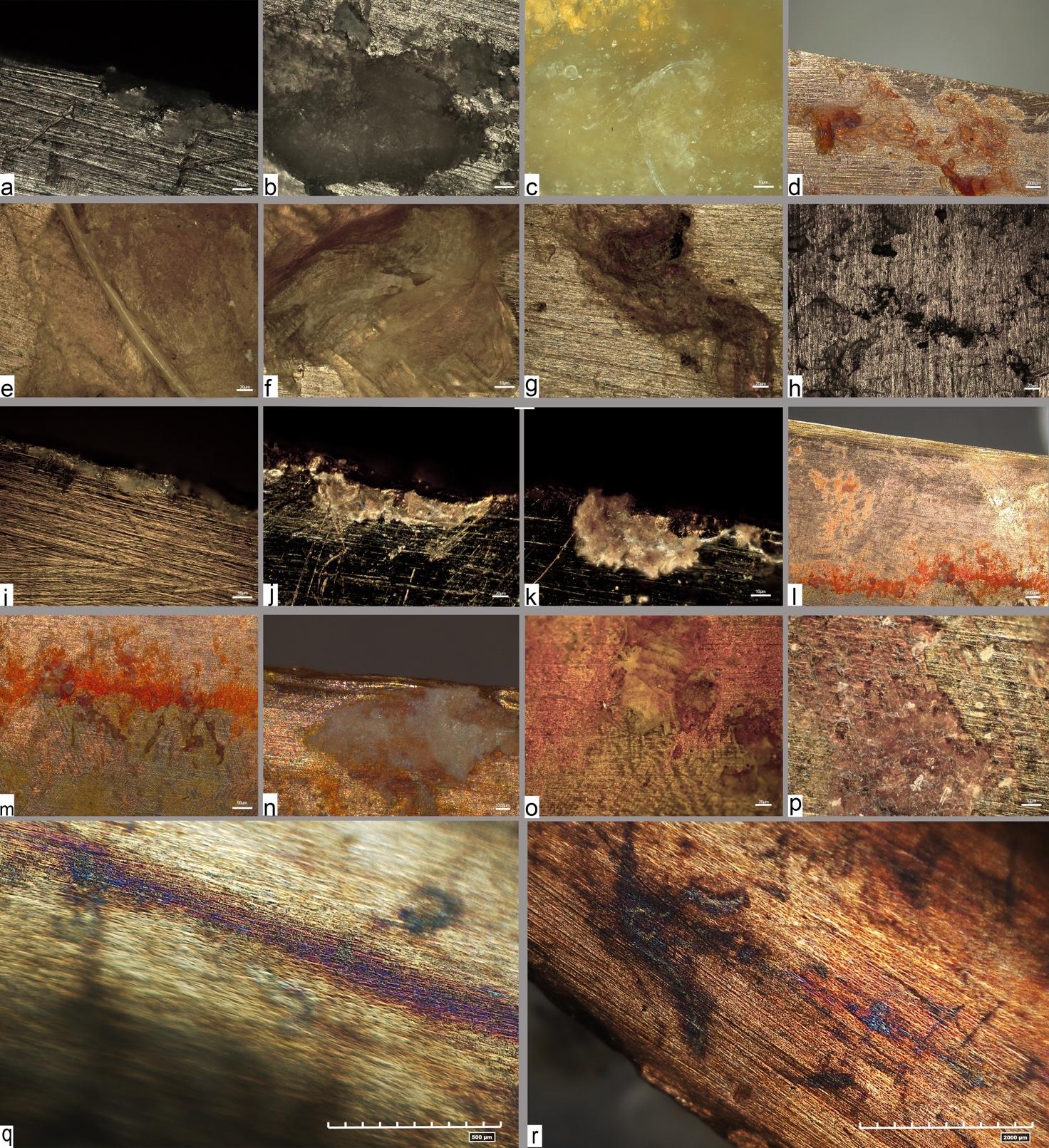


**Figure S7. Experimental residues observed under the stereomicroscope and metallographic microscope (reflected light). Animal residues: a-c)** Bone; **d)** Fat residue; **e)** Hair; **f)** Muscle fibers; **g)** Leather; **h)** black spots; **i-k)** **Siliceous plant residues**; **Wood residues: j-m)** orange to green corrosion; **n)** Amorphous residue of wood; **o)** wood fibers associated with corrosion; **p)** degraded wood residue; **q-r**) red corrosion in correspondence with animal organic residues.


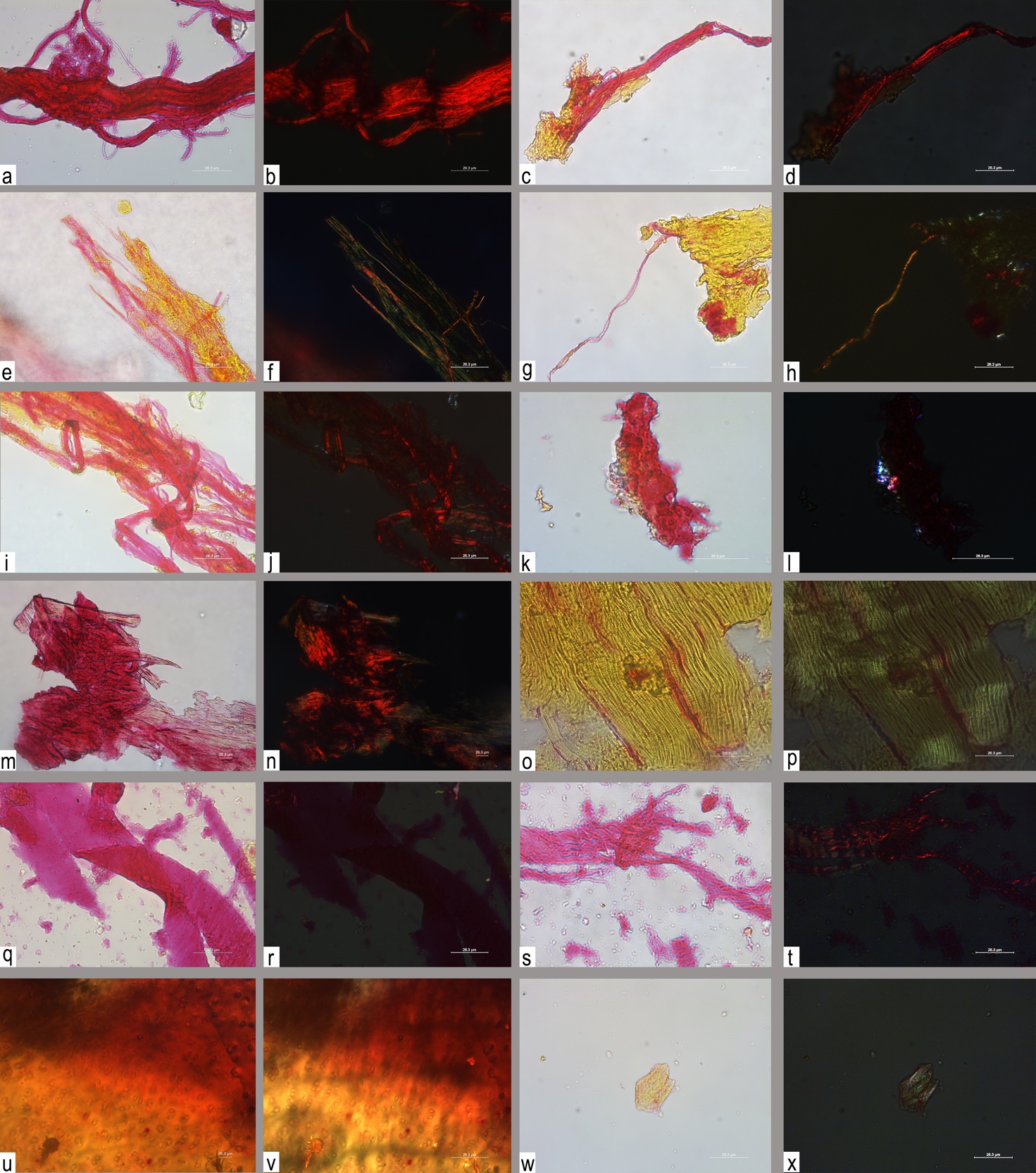


**Figure S8. Experimental animal micro-residues observed in transmitted and cross-polarized light. a-d**) tendons; **e-f**) bone fibers; **g-h**) bone periosteum and fiber; **i-j**) bone fibers; **k-l**) bone, amorphous compact residues with a rough/ cratered surface; **m-n**) Longitudinal view: overlapping fragments, angulated edges; **o-p**) skeletal muscle, striated muscle tissue, parallel reticulum of fibers; **q-t**) leather fibers; **u-v**) connective tissue, hyaline cartilage containing chondrocytes; **w-x**) amorphous collagen.


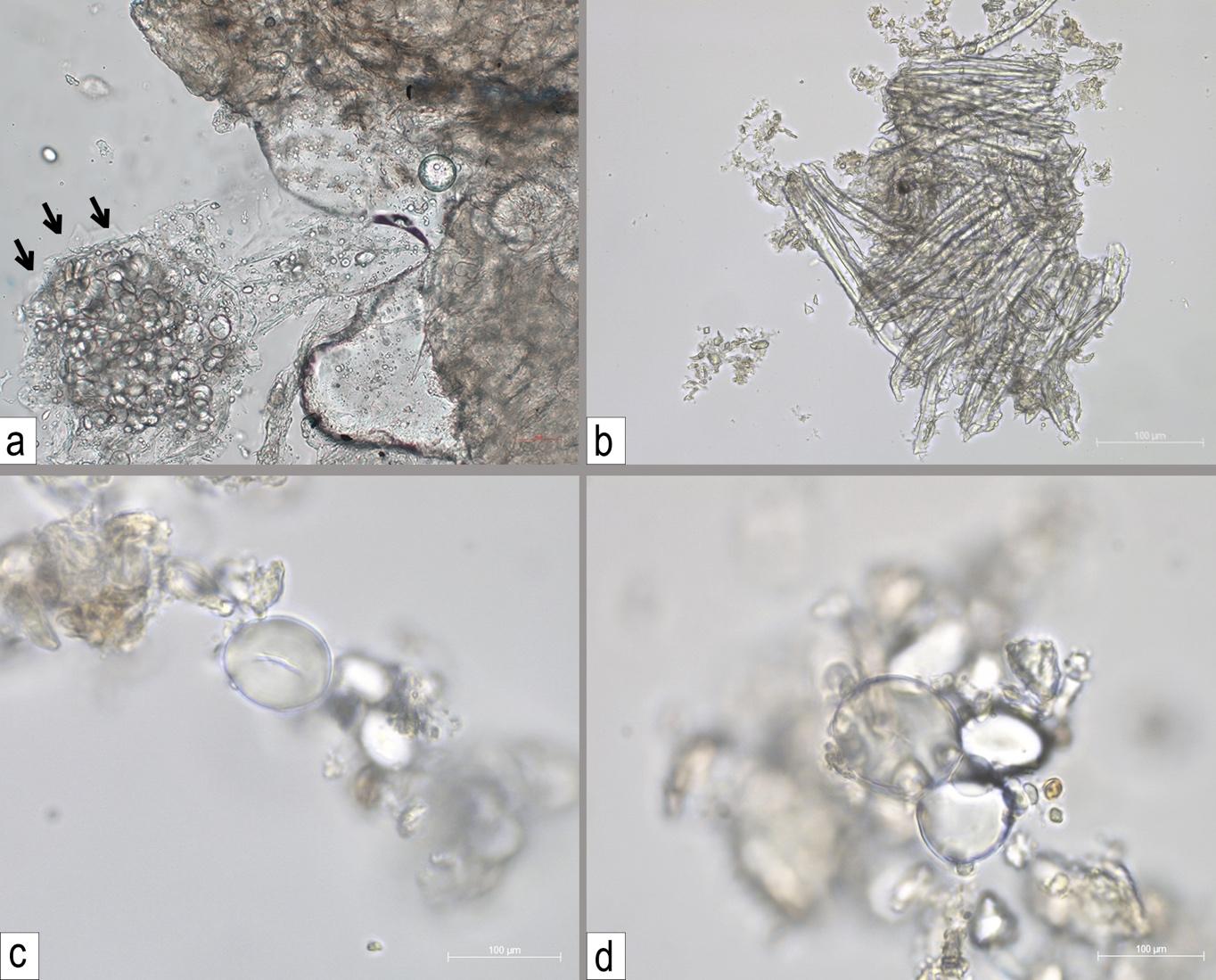


**Figure S9.** Experimental plant residues. **a**) Contamination of starch grains incurred during the butchering activity; **b**) wood fibers; **c-d**) starch grains of *Triticum monococcum*.


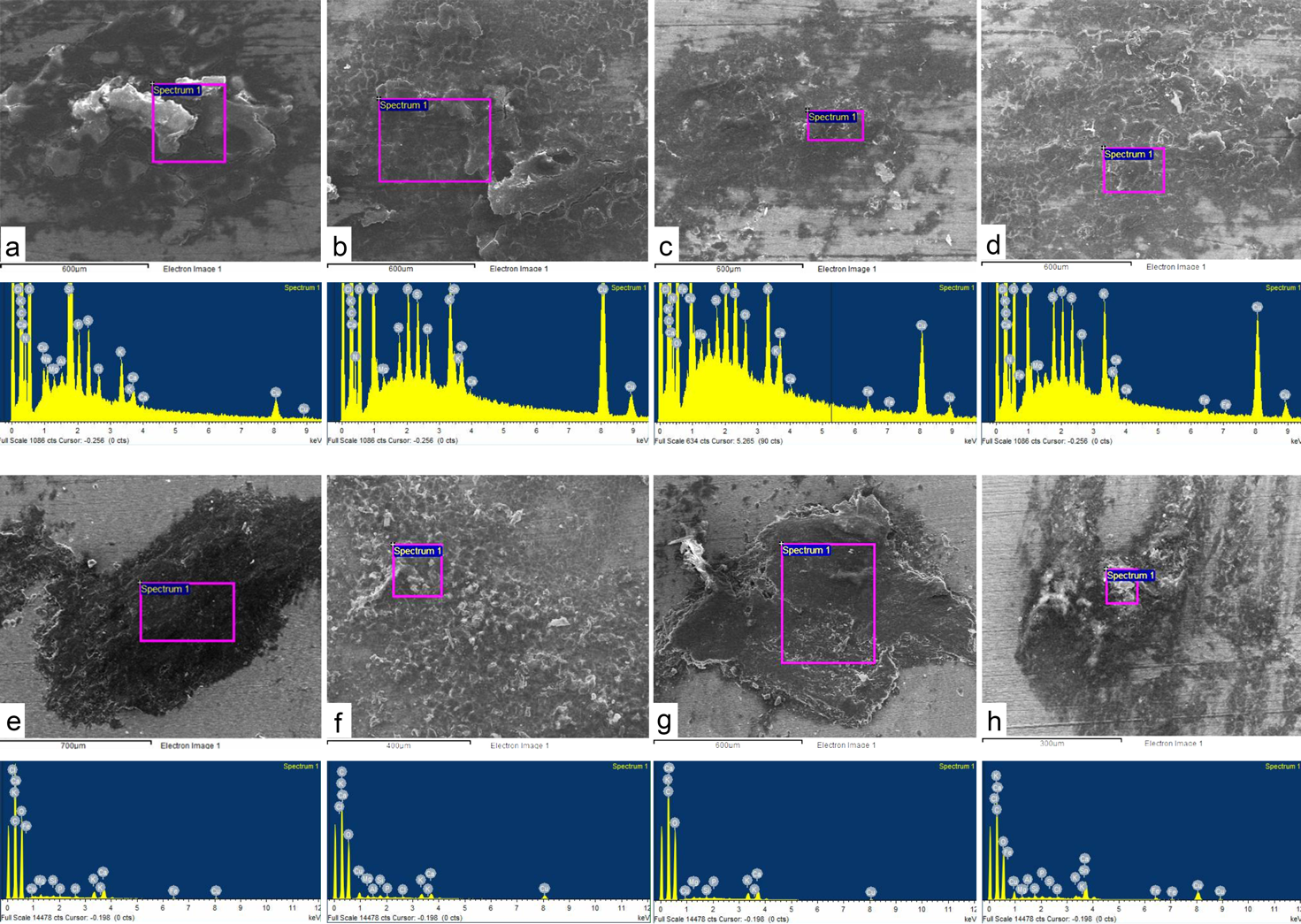


**Figure S10**. SEM-EDX analysis of experimental organic residues. **a-d**) butcher residues; **e-h**) wood residues

**Table S1.** Table with area of provenance, dimensions and description of archaeological metal dagger come from the *Terramara* of Pragatto (Bologna, Italy).

| **Dagger ID** | **Area/US** | **Length (mm)** | **Width (mm)** | **Thickness (mm)** | **Weight (g)** | **Description** | **Chronology** | **Tipology** |
| --- | --- | --- | --- | --- | --- | --- | --- | --- |
| **264** | Area A-US 114Q AU64 | 140 | 27 | 30 | 33 | Dagger with a simple base, foliate blade with central rib and n.2 rivets for the handle | (BM2) | S.Ambrogio |
| **1617** | Area B3-US 3264 Q AC96 | 110 | 21 | 30 | 18 | Dagger with incomplete base, foliate blade with central rib, n.1 residual rivet | (BM2-BM3) | Redù |
| **2037** | Area B2-US 6872 Q N112 | 85 | 23 | 30 | 19 | Dagger with incomplete base, triangular blade with central rib | (BM2-BM3) | Indeterminable |
| **2041** | Area B2-US 4065 Q D128 | 100 | 22 | 30 | 22 | Dagger with incomplete base, n.2 holes for handle, lenticular blade with central rib | (BM2-BM3) | Indeterminable |
| **1321** | Area A2-US656 Q. N40 | 150 | 20 | 60 | 30 | Dagger with a simple remodeled base, lenticular blade with a large central rib | (BM2-BM3) | Indeterminable |
| **175** | AREA A-US92 Q.AM64 | 65 | 25 | 30 | 15 | Dagger with a simple ogival base with 3 rivets for the handle, incomplete blade with a hint of central rib | (BM2-BM3) | Perteghelle |
| **1707** | Area B2-US 4065 Q AM120 | 70 | 25 | 50 | 34 | Dagger with a long tang and n.1 hole for handle | (BM3-BR) | Torre Castelluccia |
| **2035** | Area B2-US 7935 Q H120 | 150 | 21 | 40 | 46 | Rectangular tang dagger with n.2 holes for the handle and n.1 preserved rivet, foliated blade with central rib | (BM3-BR) | Campegine |
| **1683** | Area B2-US 4065 Q AM124 | 110 | 65 | 23 | 35 | "Lingua da Presa" dagger with raised edges, lenticular blade, 2 holes for handle and n.1 rivet | (BR) | Toscanella |
| **1798** | Area B2-US 4065 Q AC120 | 170 | 24 | 50 | 61 | Triangular tang with n.1 rivet, foliate blade | (BR) | Campegine |

**Table S2.** Morphological features of collagen observed on the copper-alloy archaeological daggers from Pragatto. The table lists the color (in both transmitted and cross-polarized light) and birefringence of each type of residue identified, along with residue interpretation.

| **Morphological Features of Residues** | **PSR** | | | **Residue Interpretation** |
| --- | --- | --- | --- | --- |
|  | **Color in transmitted light** | **Color in cross-polarized light** | **Birefringence** |  |
| Sheets collagen with an angular outline | Red | Red and orange/yellow with black spots | High | Bone |
| Amorphous compact residues with a rough or cratered surface and peripheral crystalline fragments | Red | Red | Medium-High | Bone |
| Tissue with longitudinal grooves (c.30 µm wide) | Red | Red | Low-Medium | Probably Bone |
| Bundles of fibers (c.1-2 µm in diameter) | Pink/Red | Red | Medium-High | Tendons |
| Striated muscle tissue (c.1.72-2 µm in diameter) | Pink/Yellow | Orange and Green | Low-Medium | Muscle |
| Amorphous matter | Yellow | Green | Low | Collagen |

**Table S3.** Description and interpretation of use and non-use related residues observed on the archaeological copper-alloy daggers from Pragatto.

| **Dagger ID** | **Site area; context; grid ref** | **Use-derived residues on cutting edges** | **Interpretation** | **Non-use residues and their location** |
| --- | --- | --- | --- | --- |
| **264** | Area A; US 114; Q AU64 | Amorphous compact residues with a rough or cratered surface and peripheral crystalline fragments | Contact with bone | Remnants of bone handle on the hafting plate |
|  |  | Bundles of fibers |  |  |
| **1617** | Area B3; US 3264; Q AC96 | Amorphous compact residues with a rough or cratered surface and peripheral crystalline fragments | Contact with bone | None |
|  |  | Amorphous collagen |  |  |
|  |  | Clumps of bone flakes with an angular outline |  |  |
| **1683** | Area B2; US 4065; Q AM124 | Amorphous compact residues with a rough or cratered surface and peripheral crystalline fragments | Contact with bone | None |
|  |  | Tissue with longitudinal grooves |  |  |
| **1707** | Area B2; US 4065; Q AM120 | sheets collagen with an angular outline | Contact with bone | Animal fur on the tang and hafting plate |
| **1798** | Area B2; US 4065; Q AC120 | Parallel reticulum of fibers | Contact with multiple tissues including muscle, bone, and tendons | None |
|  |  | Amorphous collagen |  |  |
|  |  | Amorphous compact residues with a rough or cratered surface and peripheral crystalline fragments |  |  |
|  |  | Bundles of fibers |  |  |
| **2035** | Area B2; US 7935; Q H120 | Damaged starch grains | Contamination | None |
| **2037** | Area B2; US 6872; Q N112 | Amorphous compact residues with a rough or cratered surface and peripheral crystalline fragments | Contact with multiple tissues including muscle, bone, and tendons | Mineralized plant fibers on the hafting plate and lower blade |
|  |  | Bundles of fibers |  |  |
|  |  | Damaged starch grains | Contamination |  |
| **1321** | Area A2-US656; Q N40 | Bundles of fibers | Contact with multiple tissues including muscle, bone, and tendons | Mineralized plant fibers on the hafting plate |
|  |  | Amorphous compact residues with a rough or cratered surface and peripheral crystalline fragments |  |  |

**Table S4.** List of the experiments conducted and some details on the experimental replicas.

| **Exp. Dagger** | **Type of Activity** | **Dimensions (cm)** | | | **Tin %** | **Blade**  **Description** | **Time of Use** | **Effectiveness** |
| --- | --- | --- | --- | --- | --- | --- | --- | --- |
|  |  | **Length** | **Width** | **Thickness** |  |  |  |  |
| **1** | Butchering, multiple contact with organic tissues | 7.5 | 2.5 | 0.3 | 4 | Triangular blade, simple base, 3 rivets and wooden handle | 5h | High |
| **2** | Butchering, multiple contact with organic tissues | 15.6 | 3.4 | 0.5 | 10 | Triangular blade with simple base with handle in deer antler | after 3h required sharpening | Low |
| **3** | Bone scraping and cutting | 7.5 | 2.5 | 0.3 | 4 | Triangular blade, simple base, 3 rivets and wooden handle | 3h | High |
| **4** | Fresh leather scraping and cutting | 9 | 2 | 0.6 | 10 | Triangular blade with tongue; handle in deer antler | 3h 10m | Medium |
| **5** | Tendon cutting+contact with cartilage | 7.5 | 2.5 | 0.3 | 4 | Triangular blade, simple base, 3 rivets and wooden handle | 4h | High |
| **6** | Muscle cutting | 7.5 | 2.5 | 0.3 | 10 | Triangular blade, 3 rivets and wooden handle | 5h | High |
| **7** | Cutting of cereals | 15 | 6 | 0.9 | 10 | Triangular blade with simple base with ribs; 4 nails and wooden handle | 4h 3m | Medium |
| **8** | Woodworking, both cutting, drilling and scraping | 18 | 5 | 0.5 | 10 | Triangular blade, with simple base and 4 rivets; wooden handle | 5h 30m | High |

**Table S5.** Description of the experimental macro-residues observed on replicas of bronze daggers.

| **Type of Residues** | **Residue observed** | **Macro-Residues** | | | |
| --- | --- | --- | --- | --- | --- |
|  |  | **Location** | **Color** | **Morphology or structure** | **Appearance** |
| Animal | Bone | Concentration on the proximal part of the cutting edge | White | Amorphous matter associated with translucent, striated, collagen fibers | “Bubble” appearance; striations; glossy |
|  | Fat | Proximal and internal part of the blade | Orange | Amorphous reddish matter | Glossy |
|  | Hair | Along the blade | White and Black | Cylindrical structure; different types of hair (long and white; short and black) | Glossy |
|  | Skeletal Muscle | Proximal and internal part of the blade | From white to orange/ brown | parallel reticulum of fibers | Opaque |
|  | Leather | Proximal and internal part of the blade | Brown/black | Amorphous matter | Glossy |
| Plant | Wood | Proximal and internal part of the blade | From white to orange | Dense glossy masses of white and orange wood tissue; cellulose fibers | Glossy |
| Plant | Cereals | Proximal part of the cutting edge | From brown to white | Dense opaque crust+powder | Opaque |

**Table S6.** Description of the experimental micro-residues observed on replicas of bronze daggers.

| **Micro-Residues** | **Morphological Features** | **PSR** | | |
| --- | --- | --- | --- | --- |
|  |  | **Color** | **Color in cross-polarised light** | **Birefringence** |
| Tendons | Parallel elongated bundles of cylindrical-shaped fibers (1-2 µm in diameter) | Red | Red | Medium-High |
| Bone Periosteum | Bundles of fibers or single flat fibers attached to compact bone with longitudinal fissures; fiber diameter between 1 µm to 7 µm | Pink-Red | Red; Orange/Yellow, Green | Medium-High; |
| Bone | Amorphous compact residues with a rough/ cratered surface | Red-Yellow | Red | Low-Medium |
| Bone | Longitudinal view: overlapping fragments, angulated edges | Red;  orange/yellow and black spots | Red;  orange/yellow | High |
| Skeletal muscle | Striated muscle tissue, parallel reticulum of fibers, cross striations (c. 1-2 µm in diameter) | Yellow/Green with red spot | Green | Low-Medium; |
| Leather | Thick (c.40-60 µm), amorphous and wavy fibers | Pink-Red | Red | Absent-Low |
| Leather | Bundle of fibers (c.1-2 µm in diameter) | Pink-Red | Red | Medium |
| Connective Tissue | Hyaline cartilage containing chondrocytes | Red and Orange | Red, yellow with black spots | High |
| Amorphous Collagen | Rough amorphous matter | Yellow; in some case with red spot | Green;  in some case with red spot | Low |
| Hair | Cylindrical structure; presence of medullae and sometimes the scaly cuticle or outer lay | Red-Yellow | Silver | High |

**Table S7.** Dimension of archaeological starch grains.

| **Sample** | **Starch morphotype** | **Mean Lenght (SD)** | **Range (μm)** | **n.** |
| --- | --- | --- | --- | --- |
| **2037** | Group I Type A+B | 13.6 (10.6) | 2.9-66.3 | 152 |
| **2035** | Group II Type A+B | 9.8 (5.2) | 2.6-18.6 | 29 |
| **Soil sample** | Group III Type A+B | 11.7 (12.4) | 2.6-47.9 | 67 |

**References**

1. Gozzadini G., *Il sepolcreto di Crespellano nel Bolognese* (Bologna, Tipografia Fava e Garagnani, 1881).

2. Miari M., Boccuccia P., Barbieri M., Bosi G., Carra M.L., Cremaschi M., Curci A., De Angelis A., Gabusi R., Lemorini C., Maini E., Mariani G.S., Mercuri A.M., Pavia F., Scacchetti F., Stellacci S., Gli scavi alla Terramara di Pragatto (BO): dai primi dati al progetto di ricerca, in Atti del Convegno di Studi in onore di Maria Bernabò Brea, M. Maffi, L. Bronzoni, P. Mazzieri, P., Eds. (Piacenza: Archeotravo Cooperativa Sociale, 2019), pp. 229-239.

3.Bernabò Brea M., Cremaschi M., “Le terramare: palafitte a secco o villaggi arginati?”, in *Le Terramare. La più antica civiltà padana*, M. Bernabò Brea, A. Cardarelli, M. Cremaschi, Eds. (Electa, 1997), pp. 187-195.

4.Bernabò Brea M., Cardarelli A., Cremaschi M., “Terramare. Cinque secoli di vita nella grande pianura”, in *Le Terramare. La più antica civiltà padana*, M. Bernabò Brea, A. Cardarelli, M. Cremaschi, Eds. (Electa, 1997), pp. 23-29.

5.Cupitò M., Dinamiche costruttive e di degrado del sistema aggere-fossato della Terramara di Castione dei Marchesi (Parma): rilettura e reinterpretazione dei dati ottocenteschi, in *Rivista di Scienze Preistoriche* – **LXII**, pp. 231-248 (2012).

6.Iaia C., Smiths and smithing in Bronze Age Terramare, in *Archaeology and Crafts. Experiences and Experiments on traditional Skills and Handicraft in Archaeological Open-Air Museums in Europe*, R. Kelm, Ed. (Husum: Albersdorfer Forschungen zur Archäologie und Umweltgeschichte, 2015), pp. 78-93.

7.Peroni V. B., *I pugnali nell'Italia continentale* (Prähistorische Bronzefunde: Abt. 6., 1994).

8.Pellegrini A., “Studio delle tecniche di produzione e delle modalità di utilizzo di pugnali in bronzo provenienti dalla Terramara di Pragatto (BO) Bronzo Medio e Recente”, Unpublished Master Thesis, Sapienza University of Rome, Italy (2021).

9.Dolfini A., The function of Chalcolithic metalwork in Italy: An assessment based on use-wear analysis. Journal of Archaeological Science, 38(5), pp. 1037-1049 (2011).

10.Iaia C., Dolfini A., Manufatti eneolitici in lega di rame dall’area di Roma: un’indagine su tracce di processi tecnologici e di usura, in, Roma prima del mito. Abitati e necropoli dal neolitico alla prima eta’ dei metalli nel territorio di Roma (VI-III millennio a.C.), vol. 2, A.P. Anzidei, G. Carboni, Eds. (Oxford: Archaeopress, 2020) pp. 549-568.

11.De Marinis A.M., Agnelli P., Guide to the microscope analysis of Italian mammals hairs: Insectivora, Rodentia and Lagomorpha. *Italian Journal of Zoology*, **60(2)**, pp. 225-232 (1993).

12.Debrot S., Fivaz G., Mermod C., Weber J. M., *Atlas des poils de mammifères d'Europe* (Neuchatel, Institut de Zoologie, 1982), pp. 1-208.

13.Lochte T., *Atlas der menschlichen und tierischen Haare* (Schops Verlag, Leipzig, 1938).

14.Teerink B. J., *Hair of west European Mammals* (Cambridge University Press, 1991).

15.Hammer Ø., Harper D.A.T., Ryan P. D., Past: Paleontological Statistics Software Package for Education and Data Analysis. *Palaeontologia Electronica*, **4 (1)**, pp.1-9 (2001). <http://palaeo-electronica.org/2001_1/past/issue1_01.htm>.

16.Martín-Viveros J. I., Ollé A., Using 3D digital microscopy and SEM-EDX for in-situ residue analysis: A multi-analytical contextual approach on experimental stone tools. *Quaternary International*, **569**, pp. 228-262 (2020).

17.Lynch V., Miotti L., Introduction to micro-residues analysis: systematic use of scanning electron microscope and energy dispersive X-rays spectroscopy (SEM-EDX) on Patagonian raw materials. *Journal of Archaeological Science: Reports*, **16**, pp.299-308 (2017).

18.Watson A.J., Dadswell H.E., Influence of Fiber Morphology on Paper Properties. *Appita: Technology, Innovation, Manufacturing, Environment*, ***70*(3)**, pp. 271-282 (2017).
